# Oscillating and stable genome topologies underlie hepatic physiological rhythms during the circadian cycle

**DOI:** 10.1371/journal.pgen.1009350

**Published:** 2021-02-01

**Authors:** Jérôme Mermet, Jake Yeung, Felix Naef

**Affiliations:** The Institute of Bioengineering (IBI), School of Life Sciences, Ecole Polytechnique Fédérale de Lausanne (EPFL), Lausanne, Switzerland; Universite Paris-Sud, FRANCE

## Abstract

The circadian clock drives extensive temporal gene expression programs controlling daily changes in behavior and physiology. In mouse liver, transcription factors dynamics, chromatin modifications, and RNA Polymerase II (PolII) activity oscillate throughout the 24-hour (24h) day, regulating the rhythmic synthesis of thousands of transcripts. Also, 24h rhythms in gene promoter-enhancer chromatin looping accompany rhythmic mRNA synthesis. However, how chromatin organization impinges on temporal transcription and liver physiology remains unclear. Here, we applied time-resolved chromosome conformation capture (4C-seq) in livers of WT and arrhythmic *Bmal1* knockout mice. In WT, we observed 24h oscillations in promoter-enhancer loops at multiple loci including the core-clock genes *Period1*, *Period2* and *Bmal1*. In addition, we detected rhythmic PolII activity, chromatin modifications and transcription involving stable chromatin loops at clock-output gene promoters representing key liver function such as glucose metabolism and detoxification. Intriguingly, these contacts persisted in clock-impaired mice in which both PolII activity and chromatin marks no longer oscillated. Finally, we observed chromatin interaction hubs connecting neighbouring genes showing coherent transcription regulation across genotypes. Thus, both clock-controlled and clock-independent chromatin topology underlie rhythmic regulation of liver physiology.

## Author summary

In the eukaryotic cell nucleus, DNA loops bring together gene promoters and distal regulatory elements to tune gene expression. To adapt to the 24h periodic changes in the environment, such as light-dark cycles, many organisms on the planet adopted an internal circadian clock encoded genetically. Thus, animal physiology resonates with external cycles. In mouse liver, thousands of genes, including ones belonging to the central clock, lipid and glucose metabolism, as well as detoxification are expressed with a 24h period. Here, we monitored DNA contacts across the circadian cycle involving the promoters of genes playing key roles in the daily physiology of the mouse liver. While the dynamics of some loops oscillates in sync with gene expression along the 24h day, other loops connected daily active regulatory elements to target gene promoters stably over time. Using arrhythmic mutant animals, we demonstrated that the maintenance of such stable loops goes beyond the circadian clock function. Also, we discovered a set of frequently interacting genes having coherent temporal expression regulation in wild type and clock impaired animals. Thus, both dynamic and static DNA contacts underlie the circadian transcription of mouse liver physiology.

## Introduction

Human behaviour and physiology have adapted to daily recurring inputs from the environment. Most animals including mammals have integrated a time monitoring device, known as the circadian clock, allowing them to resonate with these 24h external cues. The coupling of our internal clock with environmental light-dark cycles controls our wake-sleep rhythm but also, as illustrated here, the 3-dimensional (3D) shape of chromosomes in cells of the intact liver in mice. In mammals, the suprachiasmatic nucleus (SCN) receives light input from the retina and synchronizes peripheral organs through direct and indirect signalling [1]. The circadian clock is molecularly encoded and relies on interlocked feedback loops of gene function ticking in virtually every cell of the body [2]. In this model, BMAL1 and CLOCK transcription factors (TF) regulate the expression of their own repressors including *Period* (*Period1*, *Period2*) and *Cryptochrome* (*Cryptochrome1* and *Cryprochrome2*) genes [3]. Clock-based and organ-specific TF activities interweave to regulate tissue-specific rhythms in transcriptional programs and physiology [4,5]. For example, in mouse liver, TF binding as well as chromatin modifications and accessibility and PolII activity fluctuate genome-wide and drive the rhythmic expression of thousands of genes important for hepatic functions [6–8]. Furthermore, rhythms in post-transcriptional mechanisms can drive oscillations in the abundance and activity of gene products [9–12].

In this context, changes in chromatin topology along the 24h day emerge as a regulatory layer for temporal gene expression [10,13]. In the mammalian cell nucleus, chromatin is organized in a hierarchical network of 3D structures [14,15]. Regulatory interactions between DNA sequences, for example through a promoter-enhancer looping mechanism, mostly occur in *cis* within ~0.1 to few megabases (Mb) large topologically associating domains (TADs) [15–18]. In cultured cells, oscillatory chromatin contacts were reported only at large genomic scale, such as between a clock output gene and DNA sequences located on *trans* chromosomes [19] or with the nuclear lamina [20]. However, the latter mechanism was not observed in the mouse liver [21]. At a smaller genomic scale, promoter-enhancer loops in mouse tissues were shown to underlie temporal and tissue-specific gene transcription, for example through alternative promoter usage [5]. In fact, in mouse liver, the conformation of chromatin was captured at two opposite time points of the day genome-wide, reporting that changes in genomic interactions occurred mostly at the sub-TAD scale [22]. In addition, rhythms in promoter-enhancer looping were reported to resonate with transcriptional cycles in mouse tissues, with high contact frequency synchronized with active transcript synthesis [22–24]. Remarkably, oscillations in the formation of these loops were abolished in arrhythmic *Bmal1* KO animals, showing that the circadian clock sustained daily changes in genomic interactions [23]. Furthermore, the deletion of the daily connected *Cryptochrome1* (*Cry1*) intronic enhancer element abolished the dynamics of the loop and perturbed the *Cry1* transcription cycle (by reducing the frequency of transcriptional bursts), and eventually led to a short period phenotype of mutant animals [23]. These detailed analyses pointed out an important role of chromatin topology in the control of 24h transcription rhythms. However, it is not known whether changes in chromatin architecture systematically accompany such rhythms.

Here, we investigated temporal changes in chromatin conformation in livers of WT and *Bmal1* KO animals using 4C-seq. We identified 24h rhythms in promoter-enhancer looping synchronized with the expression of the core-clock genes *Bmal1*, *Period1* and *Period2*. Furthermore, we showed that promoters of clock output genes, representing key physiological properties of hepatocytes such as metabolite synthesis, detoxification and glucose metabolism, recruited surrounding elements resembling enhancers. Although PolII activity and chromatin marks oscillated at interacting DNA sites in WT livers, promoter-enhancer contact frequency was maintained at similar levels during day-time and night-time, corresponding to active and inactive transcription, respectively. This suggested that rhythmic transcription took place over a static and closed conformation of chromatin loops. Remarkably, in *Bmal1* KO animals, PolII activity and chromatin modifications no longer oscillated at these sites, while their interaction frequency remained stable over time and at a comparable level to WT, showing a clock-independent mechanism of DNA looping. Finally, we found a cluster of stable interactions linking a set of genes that were co-regulated across time and genotypes. Overall, these findings further our understanding on the role of chromatin architecture in circadian gene regulation in animals.

## Results

### Oscillating chromatin contacts accompany rhythmic gene transcription of core-clock repressors and activators

**Fig 1:**
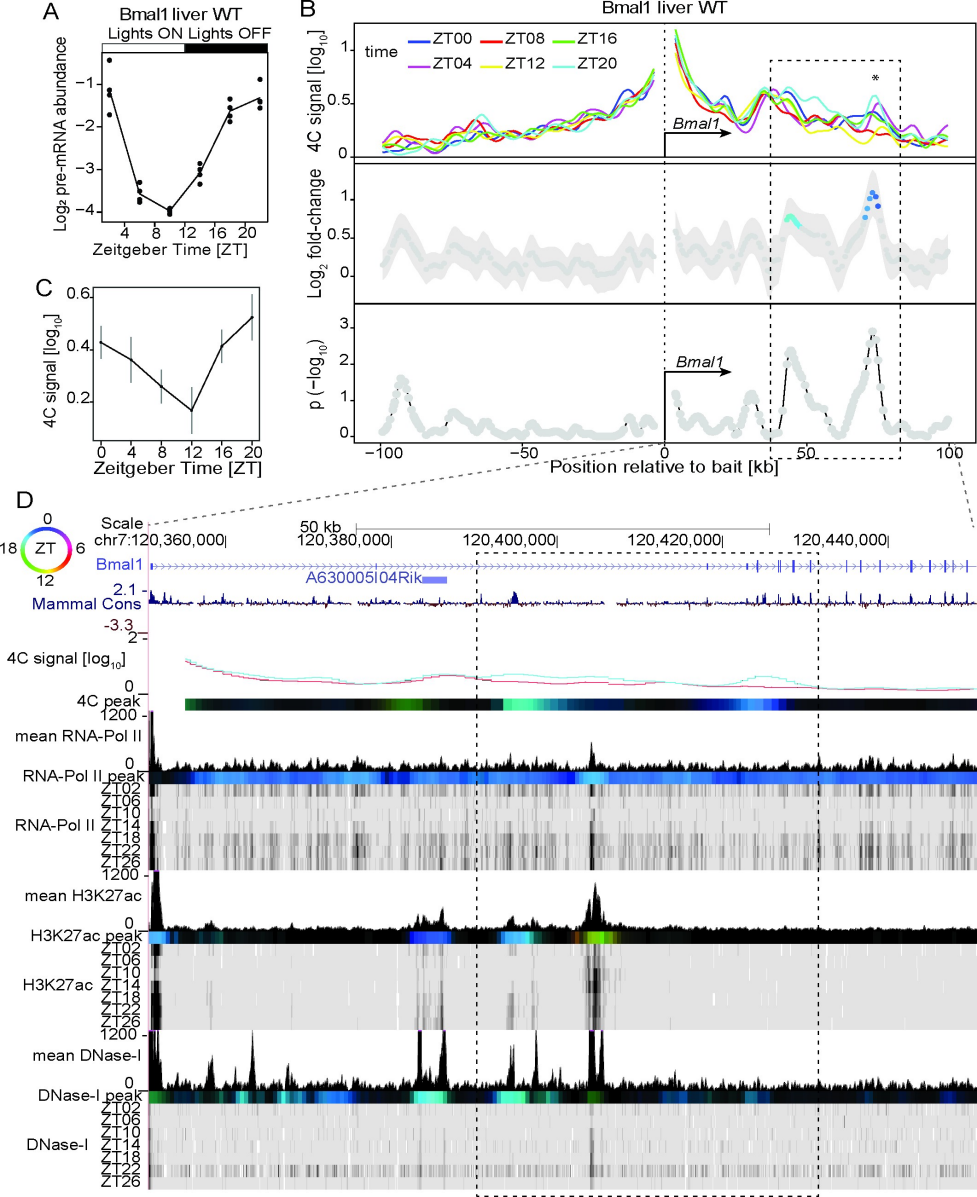
Time-resolved 4C-seq experiments revealed 24h rhythms in chromatin interactions at the *Bmal1* (*Arntl*) promoter in WT mouse liver. (A) *Bmal1* pre-mRNA expression over time in WT mouse liver [47]. (B) 4C-seq signal over time from the *Bmal1* TSS bait in WT mouse liver (top panel) and log_2_ fold change (middle panel) and **−**log_10_(p) (lower panel, Material and Methods) for rhythmicity analyses [23]. Fragments with p<0.01 are colored according to peak time in contact frequency (color-coding as in following top left circle panel D). * = local maximum in differential genomic contact. n=1 in ZT04/ZT12/ZT20, n=2 in ZT00/ZT08/ZT16. Dashed rectangle: region of rhythmic interaction. (C) 4C-seq signal over time, adjacent to * (B). (D) 4C-seq signal at ZT20 (blue) and ZT08 (red), time-resolved ChIP-seq signal for PolII, H3K27ac and DNase1 hypersensitivity in WT mouse liver [8]. Color-coded tracks represent peak time for 4C-seq signal and chromatin marks (see top left circle for time code, methods). Dashed rectangle: region of rhythmic interaction. Localized genomic regions marked by ZT18 to ZT00 H3K27ac and DNase1 hypersensitivity make chromatin contacts with the promoter region of *Bmal1* at ZT20. See S7A Fig for ChIP-seq signals of CTCF and core clock factors at the connected genomic regions.

To elucidate the dynamics of chromatin architecture along the day-night cycle in mouse tissues, we performed 4C-seq experiments every 4h for 24h as in [23] (Material and Methods). 4C-seq probes the interaction frequencies between one “bait” DNA fragment and the entire genome [25]. We placed 4C-seq baits at gene promoters of central components of the mammalian molecular clock such as *Bmal1* [3]. *Bmal1* gene is rhythmically transcribed in WT mouse liver with pre-mRNA abundance peaking around ZT22 (Fig 1A) (ZT: Zeitgeber time; ZT0 corresponds to onset of lights-on; ZT12 corresponds to onset of lights-off). Note that for circadian clock-driven gene expression, the pre-mRNA accumulation is an appropriate proxy for transcription, which typically peaks several hours before the mRNA [9]. As reported for other genes [23], the 4C-seq contacts for *Bmal1* were highly enriched on the *cis* chromosome, especially within a 2Mb region surrounding the bait position (S1A Fig). This region contained ~50% of *cis* counts for all time points (S1 Table) and comprised most gene regulatory interactions [16]. 4C-seq data were then normalized and analyzed applying a locally weighted multilinear regression (LWMR) using a Gaussian window (sigma=2.5 kb) centered on each fragment for local smoothing [23] (Material and Methods). Temporal analysis revealed that the *Bmal1* promoter rhythmically contacted a genomic region spanning from ~40 kilobases (kb) to ~75 kb downstream of the transcription start site (TSS), with the contact frequency peaking around ZT18-20 at multiple 4C-seq peaks (Figs 1B, 1C and S1A). To characterize interacting regions, we integrated time-resolved chromatin immuno-precipitation followed by high-throughput sequencing (ChIP-seq) experiments targeting PolII and chromatin marks typical of enhancer regulatory elements such as H3K27ac and DNase1 hypersensitive sites (DHS) [8]. As expected, PolII loading was rhythmic across the entire *Bmal1* gene body and peaked around ZT18-22 (Fig 1D). Multiple 4C-seq interaction sites peaking at ZT20 coincided with chromatin regions marked by rhythmic regulatory activity. For example, a preferential ZT20 contact ~45 kb downstream of the bait corresponded to a conserved intronic region marked by rhythmic H3K27ac histone acetylation and DNase1 hypersensitivity peaking around ZT20 (Fig 1D, blue region in log_2_ fold-change). Another ZT20 4C-seq peak located near exon5 ~73 kb downstream of the bait coincided with a ZT20 DNase1 signal (although weak) (Fig 1C and 1D). These data suggested oscillating DNA loops between rhythmically active enhancers and the *Bmal1* promoter.

**Fig 2:**
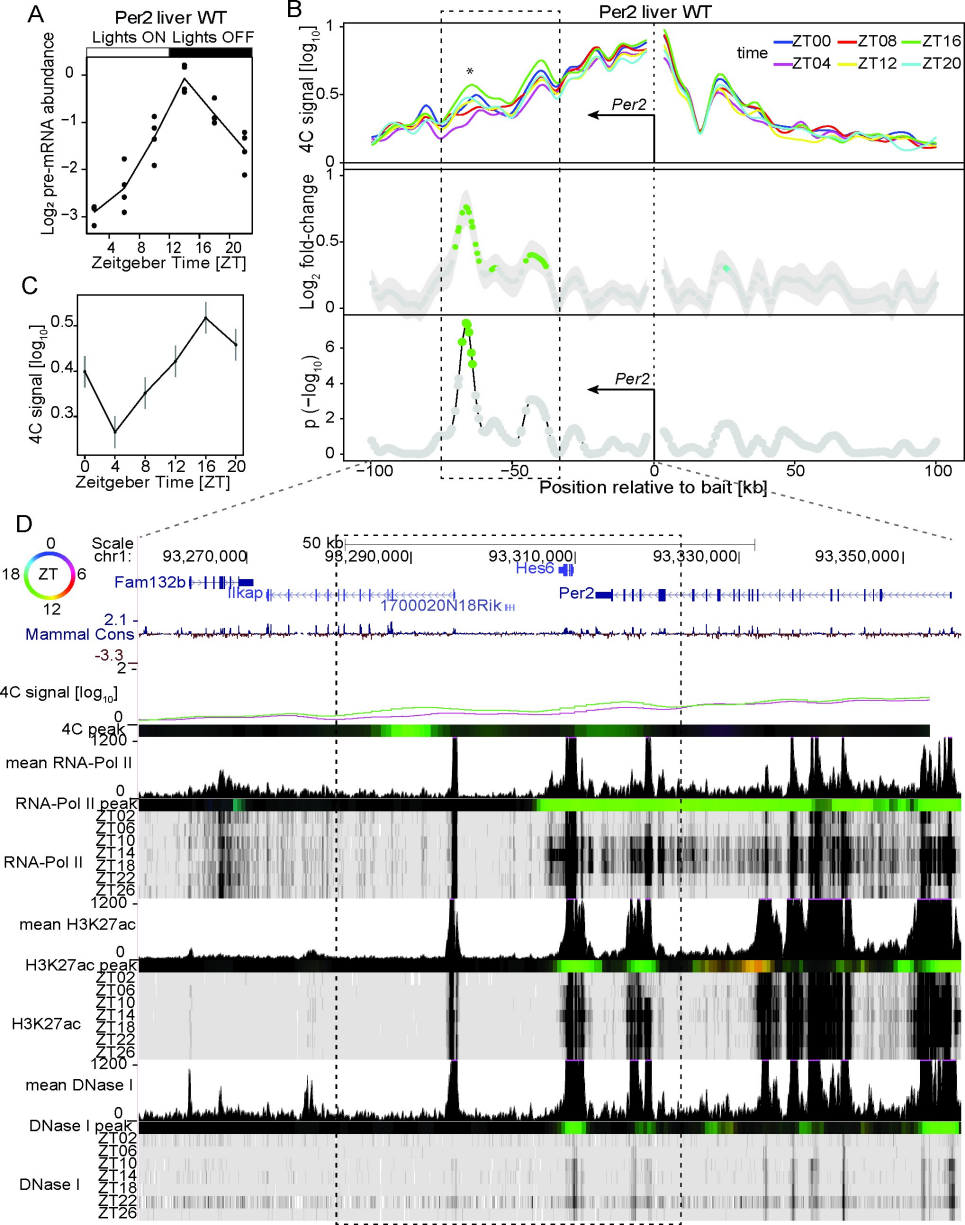
Time-resolved 4C-seq experiments revealed 24h rhythms in chromatin interactions of the *Period2* promoter in WT mouse liver. (A) *Period2* pre-mRNA expression over time in WT mouse liver [47]. (B) 4C-seq signal over time from the *Period2* TSS bait in WT mouse liver (top panel) and log_2_ fold change (middle panel) and **−**log_10_(p) from rhythmicity analyses [23]. Fragments with p< 0.01 are colored according to peak time in contact frequency (color-coding as in following top left circle panel D). * = local maximum in differential genomic contact. n=2. Dashed rectangle: region of rhythmic interaction. (C) 4C-seq signal over time adjacent to * (B). (D) 4C-seq signal at ZT04 (purple) and ZT16 (green), and time-resolved ChIP-seq signal for PolII, H3K27ac and DNase1 hypersensitivity in WT mouse liver [8]. Colored tracks represent peak time for the 4C-seq signal and chromatin marks, (top left circle for peak-time color code, Material and Methods). Dashed rectangle: region of rhythmic interaction. The region interacting with the promoter of *Period2* at ZT16 coincided with multiple localized rhythmic signals in H3K27ac and DNase1 hypersensitivity peaking at ZT16. See S7B Fig for ChIP-seq signals of CTCF and core clock factors at the connected genomic regions.

Next, we explored chromatin architecture dynamics surrounding *Period1* (*Per1*) and *Period2* (*Per2*) genes that belong to the negative limb of the circadian molecular oscillator [3]. *Per2* and *Per1* 4C-seq signals were largely enriched within a 2Mb window on *cis* chromosome (S1 Table and S1B and S1C Fig). *Per2* pre-mRNA is highest around ZT14 in WT liver (Fig 2A). At the *Per2* locus, a large region extending from ~35 kb to ~70 kb downstream of the TSS contacted more frequently the promoter at ZT16, with two prominent oscillating contacts at 40 kb and 65 kb downstream of the TSS (Fig 2B and 2C). The region 40 kb downstream of *Per2* TSS corresponded to multiple intragenic sites near the 3’ end of *Per2* containing rhythmic transcription and enhancer chromatin marks (PolII, H3K27ac, DHS) peaking around ZT16, as well as the *Hes6* gene in which PolII, H3K27ac and DHS peaked at ZT16 (Fig 2D). The region 65 kb downstream of the TSS also contained H3K27ac and DHSs enhancer marks (Fig 2D). Thus, these data showed 24h rhythms in enhancer-promoter contacts accompanying *Per2* transcription, and also dynamic gene-gene interactions with synchronised transcription. Furthermore, the *Per1* pre-mRNA level is maximal near ZT10 in WT mouse liver (S2A Fig). Overall, the chromatin rhythms showed lower amplitudes for *Per1* compared to *Per2*. While several sites within the 2Mb genomic region surrounding *Per1* showed weakly rhythmic contacts with the *Per1* promoter (S1C Fig), two proximal regions contacted the *Per1* TSS rhythmically (S2B and S2C Fig). The first region was immediately upstream of the *Per1* TSS and corresponded to multiple sites with PolII, H3K27ac and DHSs (S2D Fig). The temporal profile of this interaction showed highest contact at ZT8 (S2C Fig), but the phase of the harmonic fit was later near ZT12. The second rhythmic site located ~15-20 kb downstream of the TSS showed a peak phase near ZT14 and was in fact located in a valley of 4C signal. This site corresponded to the 3’ terminal region of *Per1* and coincided with H3K27ac and DHS sites, as well as the promoter region of *Hes7* (S2D Fig). These data suggest that sites resembling enhancer elements diurnally contacted the promoter of *Per1*. We also noted a weak ZT02 preferential interaction 45 kb upstream the *Per1* TSS (S2B Fig). Together, the measured chromatin interaction patterns at *Per1* are more complex to interpret compared to *Per2*, possibly because of weaker signals and limitations in the phase estimations.

Together, these time-resolved 4C-seq experiments revealed oscillating contacts between core-clock gene promoter and surrounding enhancers. The temporal dynamics of many of those DNA interactions as well as chromatin features at enhancer sites were synchronized with the transcription of the target gene, with high contact frequency and enhancer activity coinciding with the peak time of pre-mRNA synthesis.

### Dynamic or stable DNA loops connect daily active enhancers with the promoter of clock output genes

**Fig 3:**
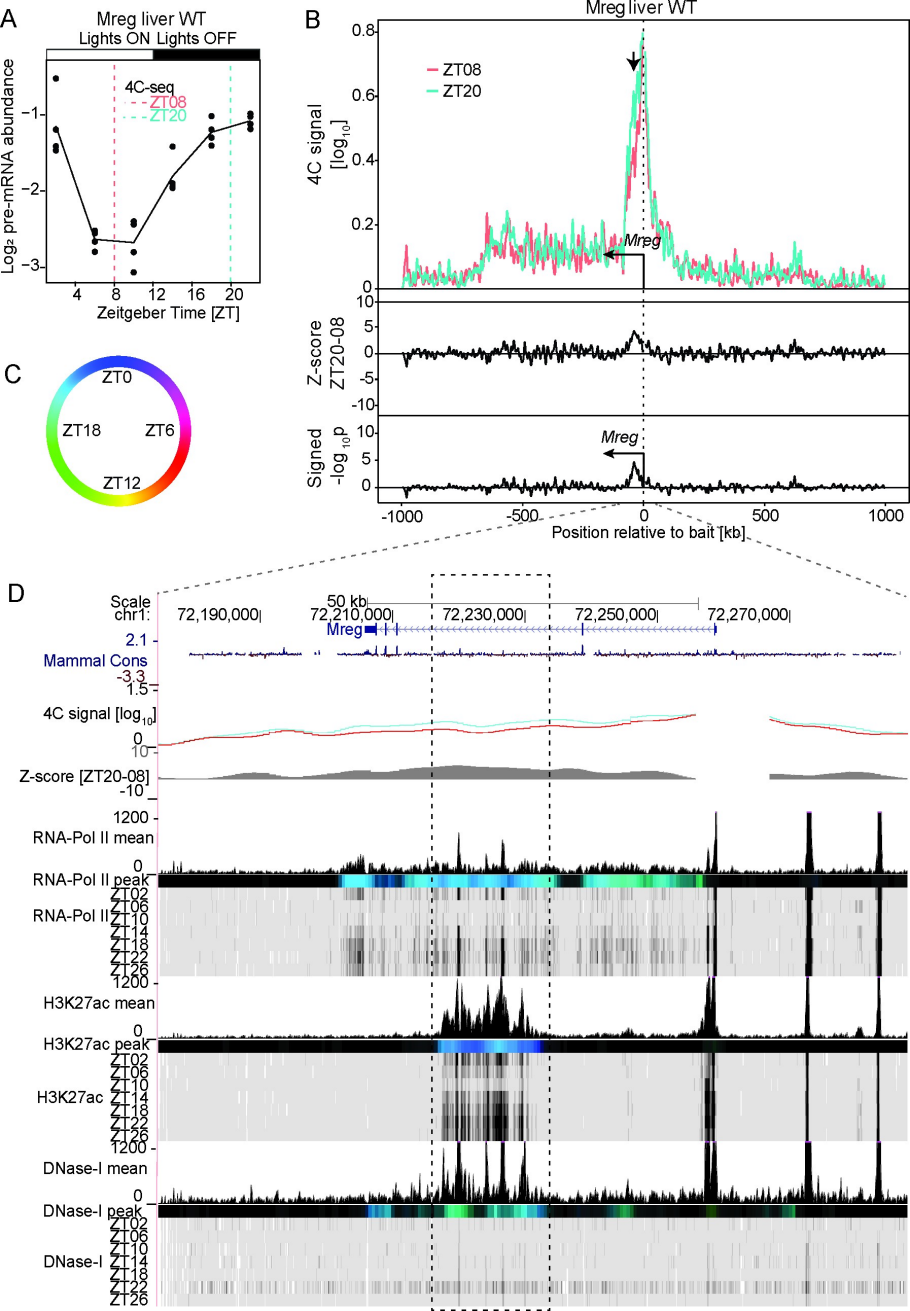
Oscillating interactions between the *Mreg* promoter and an intragenic enhancer-like elements. (A) *Mreg* pre-mRNA expression over time in WT mouse liver [47]. (B) 4C-seq signal at ZT08 (red, n=2) and ZT20 (green, n=2) in WT mouse liver in a 2Mb genomic window surrounding the *Mreg* bait position (upper panel) and the corresponding Z-scores (middle track) and p-values (lower track) revealing a ZT20 preferential contact within a region located from ~30 kb to ~40 kb downstream of the bait position (arrow). (C and D) Genome browser view with the *Mreg* 4C-seq signal at ZT08 (red) and ZT20 (blue) and time-resolved ChIP-seq signal for PolII, H3K27ac and DNase1 hypersensitivity in WT mouse liver (D) [8]. Colored tracks represent peak time in chromatin marks following the color code as in (C) (Material and Methods). Dashed rectangle: region of rhythmic interaction. The interacting region spanning from ~30 kb ~40 kb downstream of the *Mreg* TSS is marked by rhythms in DHSs and H3K27ac peaking around ZT20, in sync with *Mreg* transcription. See S8B Fig for ChIP-seq signals of CTCF and core clock factors at the connected genomic regions.

Next, we explored chromatin interactions surrounding clock output genes. In mouse liver, two main transcriptional waves centered around ZT08 and ZT20 [26] underlie daily rhythms in physiology, including detoxification [27], glucose [28] and lipid metabolism [29], and metabolite synthesis [30–32]. Notably, clock-related TFs bind to the promoter of genes involved in carbohydrate and lipid metabolism [6,33]. Thus, we selected the promoter of genes that were rhythmically transcribed (assessed by pre-mRNA peak times) with peak times that coincided with either of the two main transcriptional waves, and we profiled chromatin interactions at the two time points (ZT08 and ZT20) [26]. In particular, we placed a bait at the promoter of *Mreg* that is rhythmically transcribed specifically in the liver [5]. *Mreg* pre-mRNA peaks near ZT22 in WT mouse liver (Fig 3A). *Mreg* 4C-seq signals were enriched within the 2Mb region surrounding the bait position (S1 Table). In this signal-rich region, the 4C interaction profiles showed no difference between ZT08 and ZT20 (Fig 3B, Z-scores were centered around zero, Material and Methods) with the exception of a bait-proximal region that showed a clear preferential contact at ZT20. This region spanned from ~10 kb to ~50 kb downstream of the bait and corresponded to *Mreg* intragenic region. The ZT20 preferential contact was the highest at ~30 kb to ~40 kb downstream of the TSS (Fig 3D). Remarkably, while PolII was rhythmic and peaked around ZT20 across the entire gene body, H3K27ac and DNase1 hypersensitivity signals were high specifically within the region spanning from ~30 kb to 40 kb downstream the TSS, and both marks peaked at ZT20 (Fig 3C and 3D). This data indicated that the region recruited to the *Mreg* promoter preferentially at ZT20 corresponded to DNA elements having rhythms in enhancer chromatin signature peaking at ZT20 in WT mouse liver. Thus, oscillating DNA loops can connect daily active enhancers with promoters of clock output genes.

**Fig 4:**
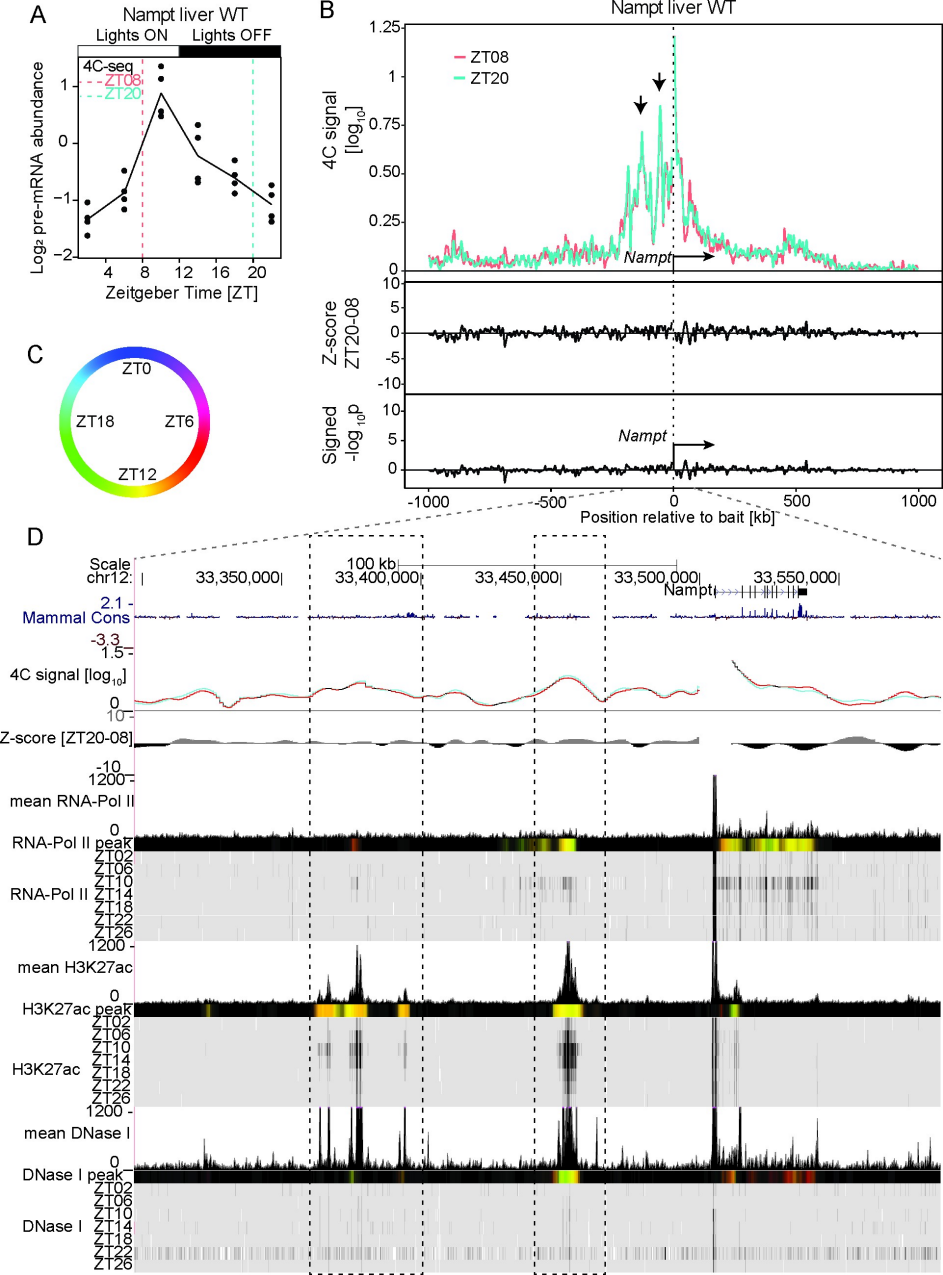
The *Nampt* promoter connects enhancer-like distal elements having rhythmic chromatin modifications. (A) *Nampt* pre-mRNA accumulation over time in WT mouse liver from [47]. (B) 4C-seq signal at ZT08 (red, n=2) and ZT20 (green, n=4) in WT mouse liver in a 2Mb genomic window surrounding the *Nampt* bait (upper panel) and the corresponding Z-scores (middle track) and p-values (lower track). The most prominent 4C-seq peaks are located ~50 kb and ~125 kb upstream of the bait position (arrows). (C and D) Genome browser view with the *Nampt* 4C-seq signal at ZT08 (red) and ZT20 (blue) and time-resolved ChIP-seq signal for PolII, H3K27ac and DNase1 hypersensitivity in WT mouse liver [8] (D). Genomic regions located 50 kb and 125 kb upstream of the *Nampt* TSS are marked with DHSs and oscillating signals in H3K27ac peaking around ZT12, in sync with the transcription of *Nampt*. Colored tracks represent peak time in 4C-seq signal and chromatin mark (color code in C, Material and Methods). Dashed rectangle: region of highest interaction frequency. See S9A Fig for ChIP-seq signals of CTCF and core clock factors at the connected genomic regions.

Next we explored the dynamics of chromatin conformation surrounding genes involved in key physiological function in liver such as *Nampt*, that encodes the nicotinamide phosphoribosyltransferase rate-limiting enzyme in the NAD biosynthesis pathway [30,31]. BMAL1 binds to the promoter of *Nampt* [6] and, the NAD+-dependent histone deacetylase SIRT1 inhibits CLOCK-BMAL1 TF activity [31], illustrating the interlocking between the clockwork machinery and the metabolic state of the cell. *Nampt* pre-mRNA accumulates rhythmically in the liver of WT mice and peak at ZT10 (Fig 4A). Again, *Nampt* 4C-seq signals were mostly confined within the 2Mb region surrounding the bait at both time points (S1 Table). Unlike the dynamics observed for core clock genes and *Mreg*, no differential interactions between ZT08 and ZT20 were found across the entire 2Mb signal-rich region (Fig 4B). In particular, major 4C-seq interaction sites stood out at 50 kb and 125 kb upstream of the bait position (Fig 4B), suggesting that these regions contacted the promoter of *Nampt* at a similar frequency during day and night. Time-resolved ChIP-seq experiments showed rhythmic loading of PolII along the *Nampt* gene body peaking at ZT10, consistently with the rhythmic accumulation of *Nampt* pre-mRNAs (Fig 4A, 4C and 4D). The two upstream interacting regions were marked by rhythmic PolII loading (although the ChIP-seq signal is weak) and H3K27ac and DHS signals peaking at ZT10 (Fig 4C and 4D). We also noted secondary 4C-seq peaks 200 kb upstream and 60 kb downstream of the *Nampt* bait at sites marked by DHS and weak H3K27ac. These data showed DNA loops connecting the promoter of *Nampt* with rhythmically active enhancers, at contact frequencies that were similar at ZT08 and ZT20. The times corresponded, respectively, to the peak and trough in transcription of *Nampt* in WT mouse liver.

Consistently with the observations at the *Nampt* locus, we identified additional cases of rhythmically active enhancer-promoter pairs forming DNA loops that were stable during active and inactive transcription at the two clock output genes *Pfkfb3* (S3 Fig) and *Mfsd2a* (S4 Fig). As shown in other model systems [34,35], this suggested that the clock-controlled transcriptional machinery can act over a frozen promoter-enhancer contact network that is insensitive to transcriptional activity.

### The *Nampt* promoter-enhancer loops are maintained in *Bmal1* knock-out animals

Next, we asked if the stable chromatin topology surrounding the rhythmically transcribed *Nampt* gene promoter was maintained in clock-impaired animals. Therefore, we profiled chromatin conformation at the *Nampt* locus in livers of *Bmal1* KO animals at ZT08 and ZT20. At the transcriptional level, *Nampt* shows lower and constant levels in livers of those arrhythmic animals compared to WT (S5A Fig). Overall, the distributions of 4C-seq signals were comparable between time points and genotypes (S1 Table). Remarkably, the *Nampt* promoter-enhancer loops were maintained at similar levels in clock-impaired mice compared to WT and constantly from ZT08 to ZT20 (S5B, S5D and S6A Figs). Furthermore, PolII loading and H3K27ac chromatin marks were overall arrhythmic and lower at connected promoter-enhancer regions in *Bmal1* KO compare to WT, consistent with pre-mRNA profiles (S5 Fig). These data suggested that despite altered transcriptional output, the static promoter-enhancer loops detected in wild-type were unaltered in *Bmal1* KO at the *Nampt* locus.

### A chromatin hub connects temporally co-transcribed genes

**Fig 5:**
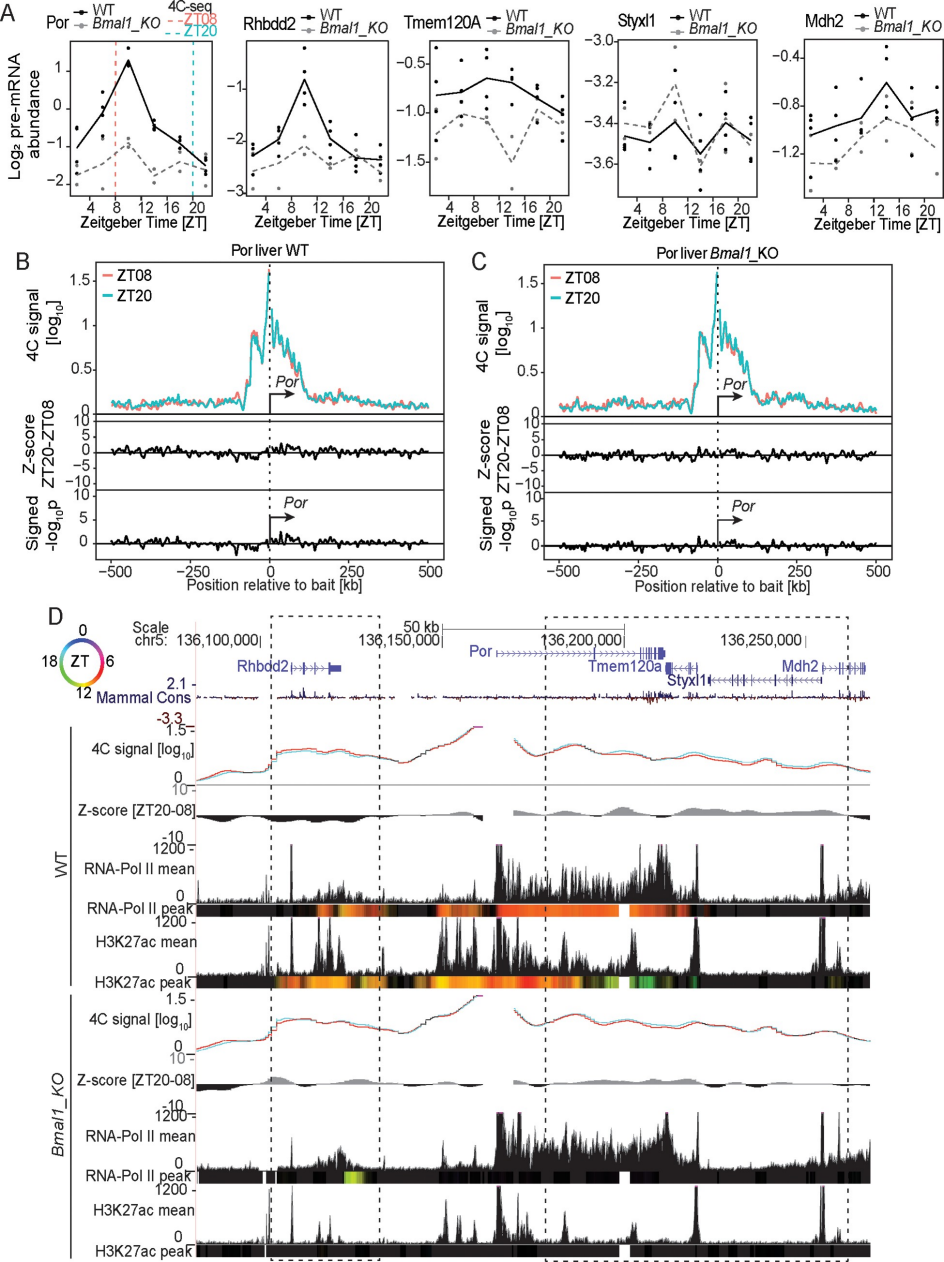
The *Por* gene stably connects surrounding genes having synchronized transcriptional dynamics in WT and *Bmal1* KO livers. (A) *Por*, *Rhbdd2*, *Tmem120a*, *Styxl1* and *Mdh2* pre-mRNAs accumulation over time in WT (solid line) and *Bmal1* KO (dashed line) livers [47]. (B and C) 4C-seq signal from *Por* TSS bait at ZT08 (red) and ZT20 (green) in a genomic window of 1Mb surrounding the bait in livers of WT (B, n=3 at ZT08 and n=4 at ZT20) and *Bmal1* KO (C, n=3) animals and the corresponding Z-scores and p-values. The most prominent interacting regions are located in the vicinity of *Por*. (D) Genome browser view of the *Por* 4C-seq signal at ZT08 (red line) and ZT20 (blue line) and the corresponding Z-scores in livers of WT and *Bmal1* KO animals. Mean and peak time of PolII and H3K27ac ChIP-seq signals are shown for both the WT and *Bmal1* KO conditions [8]. Colored tracks represent peak time in chromatin mark (color code in the top left circle, Material and Methods). Dashed rectangle: region of highest interaction frequency. PolII loading and H3K27ac oscillate in WT livers at interacting regions, notably at *Por*, *Rhbdd2*, and *Tmem120a* genes and peak around ZT10, consistently with the rhythmic transcription of these genes. The dynamics of chromatin marks is lost over the entire locus in arrhythmic *Bmal1* KO livers, while 4C-seq signals are similar to WT condition. See S9B Fig for ChIP-seq signals of CTCF and core clock factors at the connected genomic regions.

The above examples suggested that changes in transcription activity states could occur over a largely static conformation of chromatin. As shown in other systems [36,37], in such a model, gene-gene interactions might allow transcriptional co-regulation. A remarkable example arguing in favor of this model was observed at the locus of the liver-specific and rhythmically expressed gene *Por* [5]. *Por* encodes the cytochrome P450 oxidoreductase enzyme involved in the NADPH-dependent electron transport pathway; its rhythmic expression along the diurnal cycle contributes to detoxification in the mouse liver [27,38]. In WT livers, ZT08 and ZT20 4C-seq experiments using the *Por* promoter as a bait revealed chromatin contacts with a region spanning from ~50 kb upstream to ~ 100 kb downstream of the bait position, showing multiple local peaks (Fig 5B). The interaction frequency was similar between time points, as shown by the zero centered Z-scores in the *cis* region (Fig 5B). The 50 kb region upstream corresponded to *Rhbdd2* gene, while the downstream interacting region coincided with the *Tmem120a*, *Mir7034l*, *Styxl1* and *Mdh2* genes (Fig 5D). PolII loading peaked at ZT10 across the entire interacting locus, coinciding with a phase-coherent accumulation of the pre-mRNAs around ZT10 (Fig 5A and 5D), indicating that the ZT08 and ZT20 time points measured chromatin contacts during the active and inactive transcription phases, respectively. H3K27ac signals marked all prominent interacting sites, showing rhythms at the *Por* and *Rhbdd2* genes, peaking around ZT10. These data suggest that genes with synchronous transcription cycles contacted each other in WT liver, and at a constant frequency during the peak and trough of their transcription.

To further investigate the model of interaction between phase-coherent genes, we performed 4C-seq at the *Por* locus in livers of *Bmal1* KO mice. In these animals, the *Por* interacting sites were also connected at similar frequencies at ZT08 and ZT20, and at a comparable level to the WT conditions (Figs 5C and S6B), suggesting that the chromatin architecture at this locus remained stable and did not depend on a functional clock. Furthermore, *Por*, *Rhbdd2*, *Tmem120a*, *Styxl1* and *Mdh2* transcripts coherently accumulated at overall dampened, time delayed, and lower levels in *Bmal1* KO compared to WT mice (Fig 5A). Consistently, PolII loading and H3K27ac chromatin marks no longer oscillated across the entire interacting locus in livers of *Bmal1* KO compared to WT, and the levels of histone acetylation were also reduced in clock-deficient animals (Fig 5D). These data demonstrated that the *Por* interacting chromatin hub connected genes sharing similar temporal dynamics of transcription across the circadian cycle, and that the chromatin hub structure was insensitive to transcriptional changes and clock-independent.

### Chromatin loops connect distal DNA regulatory elements bound by core-clock TFs and CTCF with target gene promoters

To investigate the transcriptional regulatory function of DNA loops, we analyzed ChIP-seq data of core-clock transcription factors, in particular BMAL1, REVERB-alpha and ROR-gamma. Indeed, it was suggested that, in mouse liver, BMAL1 could connect distal regulatory elements [23,24], while REVERB-alpha, through the recruitment of co-factors, disrupts chromatin loops [22]. Furthermore, we also considered CTCF ChIP-seq data in mouse liver [39], since its role in chromatin looping is well characterized [40]. The region rhythmically recruited to the promoter of *Bmal1* (spanning from ~40 kb to ~75 kb downstream of the TSS, Fig 1B and 1D) coincided with multiple localized peaks in DNase1 hypersensitivity that were synchronous with H3K27ac rhythms, and bound by REVERB-alpha, ROR-gamma and CTCF (S7A Fig). At the *Per2* locus, we similarly observed binding of REVERB-alpha, ROR-gamma and BMAL1 and CTCF signal in the region spanning from ~35 kb to ~70 kb downstream of the TSS and containing synchronous rhythms in DNase1 hypersensitivity and H3K27ac (S7B Fig). At the *Per1* locus, BMAL1, REVERB-alpha, ROR-gamma and CTCF bound the regions rhythmically contacting the promoter (S8A Fig). These data suggested that the binding of core-clock transcription factors and CTCF at regions rhythmically recruited to the promoter of core-clock genes participate in the temporal dynamics of DNA contacts and transcription regulation. Furthermore, the rhythmic promoter-enhancer loops at the clock output gene *Mreg* were also bound by core-clock TF and CTCF (S8B Fig). At stable loops, we also observed the binding core-clock TFs and CTCF at connected sites (S9 and S10 Figs). For example, regions contacting the *Nampt* promoter were bound by REVERB-alpha and ROR-gamma (S9A Fig). At the *Por* and *Pfkfb3* loci, for which large surrounding regions connected the respective promoters, the multiple localized 4C-seq peaks coincided with ChIP-seq sites for core-clock TFs and CTCF (S9B and S10A Figs). Together, these data support a model in which binding of specific core-clock TFs as well as chromatin architecture factors at distal region connecting clock output target gene promoters participate in 24h rhythmic transcription regulation.

## Discussion

Here, we monitored chromatin contact dynamics across the 24h day at multiple core-clock and clock output gene promoters in livers of WT and arrhythmic *Bmal1* KO mice. By integrating temporal chromatin marks and transcriptomic data, we aimed at characterizing the function of chromatin topology for temporal gene expression programs and physiology. Consistently with genome-wide studies [22,24,41], we observed that both oscillating and stable genomic interactions accompanying 24h rhythms in gene expression, and found that core-clock genes show more dynamic chromatin contacts across the circadian cycle.

In a first scenario, the promoters of rhythmically transcribed genes including the core-clock genes *Bmal1*, *Period1*, *Period2* (Figs 1, 2 and S2) and clock output genes such as *Mreg* (Fig 3) recruit surrounding elements in *cis* at a specific time of the day. Such elements showed rhythms in chromatin modifications typical of regulatory enhancers, as well as binding of core-clock transcription factors and the chromatin architectural protein CTCF. Importantly, in most cases oscillations in both enhancer chromatin signatures and promoter-enhancer contact frequencies peaked in sync with the transcription of the target genes. These findings agree with other works reporting daily rhythms in promoter-enhancer looping coupled with rhythmic transcription activation [5,22–24]. Note that some chromatin interaction peaks appeared slightly delayed compared to the transcription of the target genes as for example at the site ~17 kb downstream of the *Per1* TSS (S2 Fig). While this could reflect a limitation of our experiments (4h sampling, variability in 4C-seq signals), a delay between transcription activity and chromatin looping could also reflect the nature of the genomic interaction (connecting enhancers or repressors with target gene promoters), as determined by the interplay between factors stabilizing and destabilizing DNA loops, like the transcriptional repressor REVERB-alpha and its co-factors [22]. Furthermore, we recently reported that rhythms in chromatin contact frequency depended on a functional clock [23], a mechanism that likely involves the recruitment of the mediator complex by clock TFs to connect distal sites [5,22,23]. Thus, in case of rhythmic interactions, the dynamics of DNA loops could be dominated by core-clock TFs function, allowing high-amplitude transcription [22,23]. Future work using arrhythmic animals would help at understanding, on a comprehensive scale, the role of the clock at modulating the 3-dimensional organization of the DNA along the 24h day. Intriguingly, time-resolved 4C-seq assays also revealed that *Per2* contacted the immediately downstream TF *Hes6* at ZT16, and PolII activity within *Hes6* and *Per2* was also synchronized at ZT16 and consistent with pre-mRNA accumulation (Figs 2 and S11). It may be possible that this interaction participates to the loss of *Hes6* expression rhythms in *Per1*, *Per2* double KOs [42].

In a second scenario, the promoter of rhythmically transcribed genes makes chromatin contacts with surrounding regulatory elements bound by core-clock TFs and CTCF (e.g. *Nampt*, *Pfkfb3*, *Mfsd2a*) or other genes (the *Por* interacting hub) in *cis*. Although the contacting regions displayed rhythmic chromatin modifications (in the case of regulatory elements) and rhythmic transcription (in the case of gene-gene contacts), their relative contact frequencies in the liver did not change across time, at least not between the two probed maximum and minimum transcription time-points (Figs 4, 5, S3 and S4). These data are reminiscent of other model systems in which transcription responses during development or gene induction occurs over a pre-established chromatin network [34,35]. While we measured 4C-seq contacts for the core-clock genes around the clock, for the other genes, we captured chromatin conformation at two time points coinciding with the two transcriptional waves in the liver. Though it is possible that differential genomic interaction could occur at other times of the day, the peak in transcription activity of the genes analyzed in this study, as reflected by PolII signals within gene bodies and pre-mRNA levels, reached maximum at ZT10 (or antiphasically at ZT22 for *Mreg*) and were comparatively very low at ZT18-ZT22 (Figs 4, 5, S3 and S4). Thus, the ZT08 and ZT20 4C-seq experiments most likely faithfully captured genomic contacts of the gene promoters during the highest and lowest transcription activity, respectively. Furthermore, here the chromatin contacts persisted in clock-deficient animals, coinciding with a loss of rhythm in chromatin modifications at connected regions and in transcript synthesis (Figs 5, S5 and S6). These data suggest a model in which gene promoter recruits distal regions, for example regulatory enhancers and/or co-regulated genes [36,37], forming a chromatin hub structure, over which the clock machinery regulates rhythmic mRNAs synthesis [34,35,43]. To investigate possible regulatory mechanisms, we analyzed binding of TFs at stable loop anchors and observed that, as for rhythmic interactions, core-clock TFs and CTCF bind most regions recruited stably to target gene promoters (S7–S10 Figs). Thus, the binding profiles of core-clock TFs analyzed in this study and of CTCF could not clearly differentiate dynamic from stable DNA loops. Therefore, it is likely that other factors such as clock-driven TFs contribute to the observed diversity of genomic interaction dynamics [5]. Furthermore, our data suggest that the temporal recruitment of core-clock TFs (activators and repressors) at stable DNA loop anchors is not sufficient to change the stability of pre-formed chromatin contacts.

Finally, important finding from our previous work was that deleting an intronic enhancer rhythmically recruited to the *Cry1* gene promoter shortened the period of locomotor activity rhythm in animals [23]. This effect propagated across regulatory layers, from the modulation of transcriptional bursting parameters to locomotor behavior. Here, we uncovered dozens of distal genomic regions recruited to rhythmically expressed gene promoters. While most showed chromatin signature of DNA regulatory elements, their functional contribution to transcription remains unclear. It would be interesting to evaluate if the different types of chromatin loops, for example stable versus dynamic, differentially affect transcriptional bursting parameters [44,45]. In addition, genetic manipulation might help appreciating more comprehensively the contribution of non-coding regulatory DNA to circadian biology, from transcription regulation to behavior.

## Material and methods

### Ethics statement

All experiments were approved by the Ethical Committee of the State of Vaud Veterinary Office (authorization VD3109).

### Animal housing

C57/BL6J (WT) and *Bmal1* KO animals were maintained at the EPFL animal house facility in 12hour/12hour light-dark cycle with 4 animals per cage.

### 4C-sequencing

4C-seq was performed in livers of 8 to 12 weeks old male with 4 biological replicates when comparing ZT08 versus ZT20 in WT animals and 3 biological replicates in *Bmal1* KO. 3 biological replicates were used in the 4h time-resolved 4C-seq experiments. Sample preparation and analyses were performed as in [23]. In brief, livers were isolated and perfused with PBS before homogenization in 4 mL of 1×PBS including 1.5% formaldehyde for 10 minutes at room temperature. 25 mL of the following ice-cold buffer (2.2 M sucrose, 150 mM glycine, 10 mM HEPES at pH 7.6, 15 mM KCl, 2 mM EDTA, 0.15 mM spermine, 0.5 mM spermidine, 0.5 mM DTT, 0.5 mM PMSF) was added to the homogenates and kept for 5 min on ice. Homogenates were loaded on top of 10 mL of cushion buffer (2.05 M sucrose, 10% glycerol, 125 mM glycine, 10 mM HEPES at pH 7.6,15 mM KCl, 2 mM EDTA, 0.15 mM spermine, 0.5 mM spermidine, 0.5 mM DTT, 0.5 mM PMSF) and centrifuged at 10^5^ g for 45 minutes at 4°C. Nuclei were washed twice in 1× PBS and resuspended in 1 mL of 10 mM Tris-HCL (pH 8.0), 10 mM NaCl, 0.2% NP-40, and protease inhibitor cocktail (Complete Mini EDTA-free protease inhibitor cocktail; Sigma-Aldrich); kept for 15 minutes on ice; and washed twice with DpnII buffer (New England Biolabs). Thirty million nuclei were incubated for 10 minutes at 65°C in DpnII buffer and triton X-100 was added to 1% final concentration. Chromatin was digested overnight with 400 U of DpnII (New England Biolabs) at 37°C with shaking. Digested chromatin was then diluted in 8-mL of ligation buffer containing 3000 U of T4 DNA ligase for 4 h at 16°C plus 1 h at room temperature. 50 μL of 10 mg/mL proteinase K was added and samples were incubated overnight at 65°C. DNA was precipitated and resuspended in TE buffer (pH 8.0) containing RNase A, and incubated for 30 min at 37°C. Libraries were digested with NlaIII using 1U/μg of DNA template (New England Biolabs) overnight at 37°C. Digested products were ligated with 2000 U of T4 DNA ligase (New England Biolabs) for 4 h at 16°C in a 14-mL final volume. Circularized products were precipitated and resuspended in TE buffer (pH 8.0). Inverse PCRs were performed on 600 ng of circular DNA template per sample as described in (23). Inverse PCR primers are mentioned in the S2 Table.

### 4C-sequencing analysis

4C-seq data were analyzed as in [23]. Briefly, demultiplexed read counts were mapped to the mouse genome (mm9) using HTSstation [46]. Samples were excluded from the analysis if more than 75% of restriction fragments did not have any count on a 2Mb region surrounding bait fragment (S1 Table). The first five NlaIII fragments upstream and downstream of the bait were excluded in the analysis since they were suspected to be partially digested or self-ligated products. The 4C-seq signal was calculated using a locally weighted multilinear regression model [23]. Fragment counts for each sample were normalized by the total fragments on the *cis*-chromosome (excluding the five fragments upstream and downstream of the bait). To stabilize variance, the fragment counts *c* in each sample were log-transformed:

$$Y=\log_{10}\left(\frac{c}{p}+1\right)$$

with p=500. For each position, the 4C-seq signals (*Y*) were modeled with fragment effects *a*_*i*_ and condition effects *b*_*j*_ (which can be time, tissue, or genotype). We estimated these effects by minimizing the weighted sum *S* of squared residuals across replicates *r*:

$S=\arg\;\underset{a,b}{\min}\;\sum_{i,j,r}W_{i,j}{\left(Y_{i,j,r}-a_{i}-b_{j}\right)}^{2},$ with weights *W*_*i*,*j*_ are defined as *W*_*i*,*j*_ = *w*_*g*,*i*_×*w*_*s*,*j*_, where *w*_*g*,*i*_ is the Gaussian smoothing kernel (sigma=2500bp) at position *i*, and *w*_*s*,*j*_ a condition weight based on the number of samples with non-zero counts on fragment *i*. To compare between two conditions, we calculated p-values and condition effects at each genomic fragment using t-statistics. To detect rhythmic signal, we calculated the 24-hour Fourier coefficients of the condition effect (real and imaginary parts) from the six equally-spaced time points and used the chi-square test to test deviations from the null model that the real and imaginary parts have both a mean of zero.

### RNA-seq

Processed RNA-seq data were downloaded from [47] (GSE73554) and rhythms were analyzed as in [5].

### H3K27ac and PolII ChIP-seq and DNase1-seq

Data were downloaded from [8] (GSE60430) and analyzed as in [23]. We binned the ChIP-seq and DNase1-seq signal (log2 counts per million) into 500 bp windows. We smoothed the signal by taking a running average across 7 bins (3 bins upstream and 3 bins downstream of the current bin). For each bin, we calculated the amplitude and phase by fitting a harmonic regression model with a 24-hour period across the 7 bins. The rhythmic signal (amplitude and phase) was mapped to a color using hue (time of maximum signal), saturation (set to 1), and value (increased with increasing statistical significance) color scheme.

To obtain smooth color transitions, the value *v* was calculated using a Hill function with Hill coefficient *n* = 5 and $v=\min_{i\in (a,p)}\left(\frac{{-\log\left(x_{i}\right)}^{5}}{{k_{i}}^{5}{-\log\left(x_{i}\right)}^{5}}\right)$, with *k*_*a*_ = 0.5, *k*_*p*_ = 4.5 and *x*_*a*_, *x*_*p*_ being amplitude and –log_10_(p) of the harmonic regression fit.

### REVERB-alpha, ROR-gamma and CTCF ChIP-seq

BMAL1 ChIP-seq data were downloaded from [6] (GS26602). REVERB-alpha and ROR-gamma ChIP-seq data were downloaded from [48] (GSE67973). CTCF ChIP-seq data were downloaded from [39].

## Supporting information

S1 Fig (A) 2Mb genomic window view of 4C-seq signal over time from the *Bmal1* TSS bait in WT mouse liver (top panel) and log_2_ fold change (middle panel) and **−**log_10_(p) (lower panel, Material and Methods) for rhythmicity analyses [23]. Fragments with p<0.01 are colored according to peak time in contact frequency (color-coding as top left circle in Fig 1D). n=1 in ZT04/ZT12/ZT20, n=2 in ZT00/ZT08/ZT16. (B) 2Mb genomic window view of 4C-seq signal over time from the *Per2* TSS bait in WT mouse liver (top panel) and log_2_ fold change (middle panel) and **−**log_10_(p) (lower panel, Material and Methods) for rhythmicity analyses [23]. Fragments with p<0.01 are colored according to peak time in contact frequency (color-coding as top left circle in Fig 2D). n=2. (C) 2Mb genomic window view of 4C-seq signal over time from the *Per1* TSS bait in WT mouse liver (top panel) and log_2_ fold change (middle panel) and **−**log_10_(p) (lower panel, Material and Methods) for rhythmicity analyses [23]. Fragments with p<0.01 are colored according to peak time in contact frequency (color-coding as top left circle S2D Fig). n=2.
(TIF)

S2 Fig Time-resolved 4C-seq using the *Period1* TSS as bait revealed 24h rhythmic chromatin interactions in WT mouse liver. (A) *Period1* pre-mRNA expression over time in WT mouse liver [47]. (B) 4C-seq signal over time for the *Period1* TSS bait in WT mouse liver (top panel) and log_2_ fold-change (middle panel) and **−**log_10_(p) (lower panel, Material and Methods) from rhythmicity analyses [23] (n=2). Although the 4C-seq signals were weak for the *Period1* bait, two localized genomic regions at ~6 kb upstream and ~17 kb downstream of the bait position were recruited preferentially at ZT12 to the *Period1* promoter. Fragments with p< 0.01 are colored according to peak time in contact frequency (color-coding as in following top left circle panel D). * = local maximum in differential genomic contact. n=2. Dashed rectangle: region of rhythmic interaction. (C) 4C-seq signal over time adjacent to * (B). (D) *Period1* 4C-seq signal at ZT04 (purple) and ZT12 (yellow) and time-resolved ChIP-seq signals for PolII, H3K27ac and DNase1 hypersensitivity in WT mouse liver [8]. Colored tracks represent peak time in 4C-seq signal and chromatin marks following the color code as in (C) (Material and Methods). Dashed rectangle: region of rhythmic interaction. The regions located 6 kb upstream and 17 kb downstream of the bait coincided with multiple localized peaks in H3K27ac and DNase1 hypersensitivity. See S8A Fig for ChIP-seq signals of CTCF and core clock factors at the connected genomic regions.
(TIFF)

S3 Fig The promoter of *Pfkfb3* connects enhancer-like distal elements showing rhythmic chromatin modifications. (A) *Pfkfb3* pre-mRNA expression over time in WT mouse liver [47]. (B) 4C-seq signal at ZT08 (red, n=3) and ZT20 (green, n=4) in WT mouse liver in a 2Mb genomic window surrounding the *Pfkfb3* bait position (upper panel) and the corresponding Z-scores (middle track) and p-values (lower track) revealing multiple prominent 4C peaks. Dashed rectangle: region of highest interaction frequency. (C and D) Genome browser viewing with *Pfkfb3* 4C-seq signal at ZT08 (red) and ZT20 (blue) and time-resolved ChIP-seq signal for PolII, H3K27ac and DNase1 hypersensitivity in WT mouse liver (D) [8]. Colored tracks represent peak time in chromatin marks following the color code as in (C) (Material and Methods). Dashed rectangle: region of highest interaction frequency. Multiple regions interacting with the *Pfkfb3* TSS are marked by DHSs and rhythmic H3K27ac signals peaking around ZT10, in sync with *Pfkfb3* transcription. See S10A Fig for ChIP-seq signals of CTCF and core clock factors at the connected genomic regions.
(TIFF)

S4 Fig The promoter of *Mfsd2a* connects enhancer-like distal elements showing rhythmic chromatin modifications. (A) *Mfsd2a* pre-mRNA expression over time in WT mouse liver [47]. (B) 4C-seq signal at ZT08 (red, n=3) and ZT20 (green, n=3) in WT mouse liver in a 2Mb genomic window surrounding the *Mfsd2a* bait position (upper panel) and the corresponding Z-scores (middle track) and p-values (lower track) revealing a localized prominent 4C-seq peak (although with low 4C-seq signals) ~15 kb upstream of the bait position (arrow). (C and D) Genome browser viewing with *Mfsd2a* 4C-seq signal at ZT08 (red) and ZT20 (blue) and time-resolved ChIP-seq signal for PolII, H3K27ac and DNase1 hypersensitivity in WT mouse liver (D) [8]. Dashed rectangle: region of highest interaction frequency. The genomic region located ~15 kb upstream of the bait position is marked by DHSs and rhythmic H3K27ac signals peaking around ZT12, consistently with *Mfsd2a* transcription. Colored tracks represent peak time in chromatin marks following the color code as in (C) (Material and Methods). See S10B Fig for ChIP-seq signals of CTCF and core clock factors at the connected genomic regions.
(TIFF)

S5 Fig *Nampt* promoter-enhancer interactions do not depend on BMAL1. (A) *Nampt* pre-mRNA accumulation over time in WT (solid line) and *Bmal1* KO (dashed line) livers [47]. (B) *Nampt* 4C-seq signal at ZT08 (red, n=3) and ZT20 (green, n=3) in a genomic window of 2Mb surrounding the bait in the liver of *Bmal1* KO animals and the corresponding Z-scores and p-values. (C and D) Genome browser view of the *Nampt* 4C-seq signal and the corresponding Z-scores in livers of WT and *Bmal1* KO animals at ZT08 (red line) and ZT20 (blue line) (D). Mean and peak time of PolII and H3K27ac ChIP-seq signals are shown for both WT and *Bmal1* KO conditions (D) [8]. Colored tracks represent peak time in chromatin marks following the color code as in (C) (Material and Methods). Dashed rectangle: region of highest interaction frequency in WT and *Bmal1_KO* animals. H3K27ac rhythms are observed at connected regions in WT livers. In the arrhythmic *Bmal1* KO livers, the chromatin marks no longer oscillate while chromatin contacts are maintained at levels comparable to WT conditions.
(TIFF)

S6 Fig (A) *Nampt* 4C-seq signal at ZT08 (red) and ZT20 (green) in a genomic window of 2Mb surrounding the bait in the liver of WT (n=2 at ZT08, n=4 at ZT20, dashed lines) and *Bmal1* KO animals (n=3, solid lines) and the corresponding Z-scores and p-values. *Nampt* 4C-seq signals are very similar across all conditions. (B) 4C-seq signal from *Por* TSS bait at ZT08 (red) and ZT20 (green) in a genomic window of 1Mb surrounding the bait in livers of WT (n=3 at ZT08 and n=4 at ZT20, dashed lines) and *Bmal1*_KO animals (n=3, solid lines) and the corresponding Z-scores and p-values. *Por* 4C-seq signals are very similar across all conditions.
(TIFF)

S7 Fig (A) 4C-seq signal and ChIP-seq signal for PolII, H3K27ac and DNase1 hypersensitivity in WT mouse liver at *Bmal1* locus as in Fig 1D, as well as ChIP-seq signal against BMAL1 in mouse liver at ZT06 [6], against REVERB-alpha and ROR-gamma in mouse liver at ZT10 and ZT22 respectively [48], and CTCF in mouse liver [39]. Dashed rectangle: genomic regions contacting *Bmal1* promoter preferentially between ZT18 to ZT00 are marked by synchronous rhythms in H3K27ac and DNase1 hypersensitivity as well as binding of REVERB-alpha, ROR-gamma and CTCF. (B) 4C-seq signal and ChIP-seq signal for PolII, H3K27ac and DNase1 hypersensitivity in WT mouse liver at *Per2* locus as in Fig 2D, as well as ChIP-seq signal against BMAL1 in mouse liver at ZT06 [6], against REVERB-alpha and ROR-gamma in mouse liver at ZT10 and ZT22 respectively [48], and CTCF in mouse liver [39]. Dashed rectangle: genomic regions contacting *Per2* promoter preferentially at ZT16 are marked by synchronous rhythms in H3K27ac and DNase1 hypersensitivity as well as binding of REVERB-alpha, ROR-gamma and CTCF, as well as low binding of BMAL1.
(TIFF)

S8 Fig (A) 4C-seq signal and ChIP-seq signal for PolII, H3K27ac and DNase1 hypersensitivity in WT mouse liver at *Per1* locus as in S2 Fig, as well as ChIP-seq signal against BMAL1 in mouse liver at ZT06 [6], against REVERB-alpha and ROR-gamma in mouse liver at ZT10 and ZT22 respectively [48], and CTCF in mouse liver [39]. Dashed rectangle: genomic regions rhythmically contacting *Per1* promoter are marked by peaks of H3K27ac and DNase1 hypersensitivity as well as binding of BMAL1, REVERB-alpha, ROR-gamma and CTCF. (B) 4C-seq signal and ChIP-seq signal for PolII, H3K27ac and DNase1 hypersensitivity in WT mouse liver at *Mreg* locus as in Fig 3, as well as ChIP-seq signal against BMAL1 in mouse liver at ZT06 [6], against REVERB-alpha and ROR-gamma in mouse liver at ZT10 and ZT22 respectively [48], and CTCF in mouse liver [39]. Dashed rectangle: genomic regions contacting *Mreg* promoter preferentially at ZT20 are marked by synchronous rhythms in H3K27ac, DHS as well as binding of REVERB-alpha, ROR-gamma and CTCF.
(TIFF)

S9 Fig (B) 4C-seq signal and ChIP-seq signal for PolII, H3K27ac and DNase1 hypersensitivity in WT mouse liver at *Nampt* locus as in Fig 4, as well as ChIP-seq signal against BMAL1 in mouse liver at ZT06 [6], against REVERB-alpha and ROR-gamma in mouse liver at ZT10 and ZT22 respectively [48], and CTCF in mouse liver [39]. Dashed rectangles: genomic regions contacting *Nampt* promoter are marked by rhythms in PolII, H3K27ac and DNase1 hypersensitivity as well as binding of REVERB-alpha and ROR-gamma. (B) 4C-seq signal and ChIP-seq signal for PolII, H3K27ac and DNase1 hypersensitivity in WT mouse liver at *Por* locus as in Fig 5, as well as ChIP-seq signal against BMAL1 in mouse liver at ZT06 [6], against REVERB-alpha and ROR-gamma in mouse liver at ZT10 and ZT22 respectively [48], and CTCF in mouse liver [39]. Dashed rectangles: genomic regions contacting *Por* promoter are marked rhythms in PolII, H3K27ac and DNase1 hypersensitivity as well as binding of REVERB-alpha, ROR-gamma and CTCF.
(TIFF)

S10 Fig (A) 4C-seq signal and ChIP-seq signal for PolII, H3K27ac and DNase1 hypersensitivity in WT mouse liver at *Pfkfb3* locus as in S3D Fig, as well as ChIP-seq signal against BMAL1 in mouse liver at ZT06 [6], against REVERB-alpha and ROR-gamma in mouse liver at ZT10 and ZT22 respectively [48], and CTCF in mouse liver [39]. Dashed rectangle: genomic regions contacting *Pfkfb3* promoter are marked by rhythms in H3K27ac and DNase1 hypersensitivity as well as binding of BMAL1, REVERB-alpha, ROR-gamma and CTCF. (B) 4C-seq signal and ChIP-seq signal for PolII, H3K27ac and DNase1 hypersensitivity in WT mouse liver at *Mfsd2a* locus as in S4D Fig, as well as ChIP-seq signal against BMAL1 in mouse liver at ZT06 [6], against REVERB-alpha and ROR-gamma in mouse liver at ZT10 and ZT22 respectively [48], and CTCF in mouse liver [39]. Dashed rectangle: genomic regions contacting *Mfsd2a* promoter are marked rhythms in PolII, H3K27ac and DNase1 hypersensitivity as well as binding of REVERB-alpha and ROR-gamma, and low binding of CTCF.
(TIFF)

S11 Fig *Hes6* pre-mRNAs accumulate in sync with *Per2* transcripts (Fig 2) in WT livers. Data from [47].
(TIFF)

S1 Table The table contains total 4C-sequencing read counts for each sample and the number and proportion of reads on *trans* and *cis* chromosome as well as on a 2Mb genomic region surrounding the bait fragment. (XLSX)

S2 Table Table contains sequence of inverse-PCR primers used to generate 4C-seq libraries. (XLSX)
