# Supplementary figures and images for: Oscillating and stable genome topologies underlie hepatic physiological rhythms during the circadian cycle
Source: PLoS Genet. 2021 Feb 1;17(2):e1009350. doi: 10.1371/journal.pgen.1009350 (PMC7877755; doi:10.1371/journal.pgen.1009350)

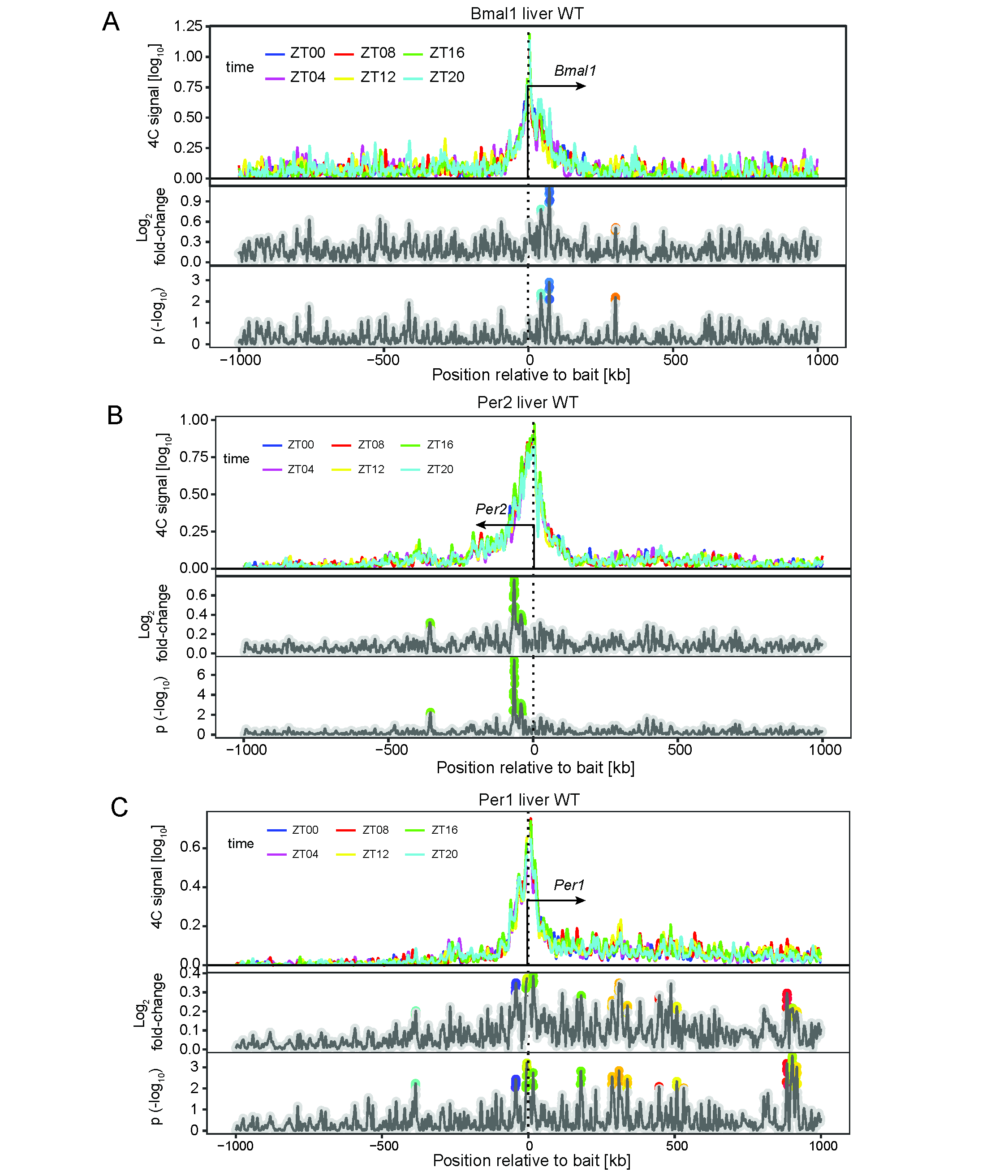

Supplement: S1 Fig — (A) 2Mb genomic window view of 4C-seq signal over time from the Bmal1 TSS bait in WT mouse liver (top panel) and log2 fold change (middle panel) and −log10(p) (lower panel, Material and Methods) for rhythmicity analyses [23]. Fragments with p<0.01 are colored according to peak time in contact frequency (color-coding as top left circle in Fig 1D). n=1 in ZT04/ZT12/ZT20, n=2 in ZT00/ZT08/ZT16. (B) 2Mb genomic window view of 4C-seq signal over time from the Per2 TSS bait in WT mouse liver (top panel) and log2 fold change (middle panel) and −log10(p) (lower panel, Material and Methods) for rhythmicity analyses [23]. Fragments with p<0.01 are colored according to peak time in contact frequency (color-coding as top left circle in Fig 2D). n=2. (C) 2Mb genomic window view of 4C-seq signal over time from the Per1 TSS bait in WT mouse liver (top panel) and log2 fold change (middle panel) and −log10(p) (lower panel, Material and Methods) for rhythmicity analyses [23]. Fragments with p<0.01 are colored according to peak time in contact frequency (color-coding as top left circle S2D Fig). n=2. (TIF) [file pgen.1009350.s001.tif]

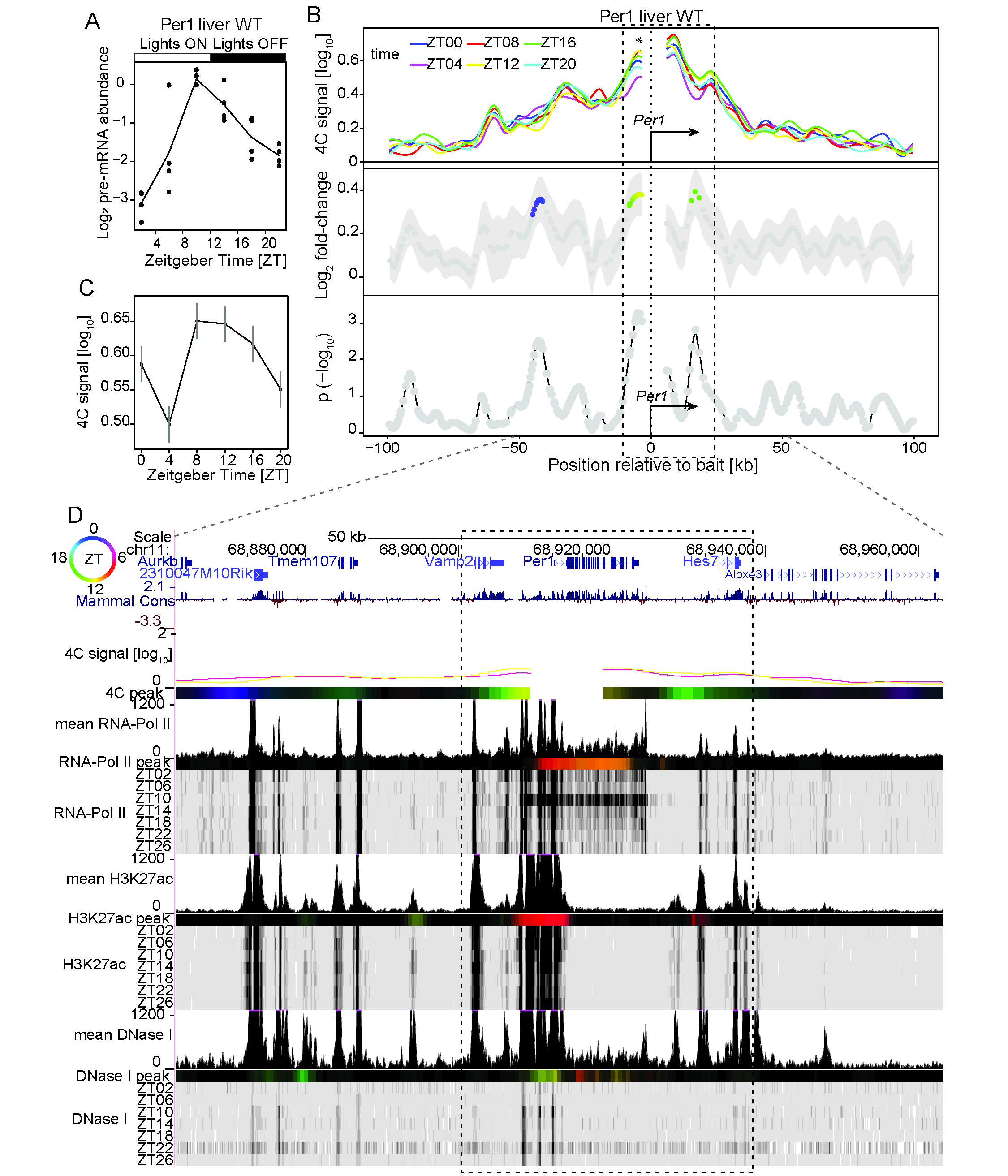

Supplement: S2 Fig — (A) Period1 pre-mRNA expression over time in WT mouse liver [47]. (B) 4C-seq signal over time for the Period1 TSS bait in WT mouse liver (top panel) and log2 fold-change (middle panel) and −log10(p) (lower panel, Material and Methods) from rhythmicity analyses [23] (n=2). Although the 4C-seq signals were weak for the Period1 bait, two localized genomic regions at ~6 kb upstream and ~17 kb downstream of the bait position were recruited preferentially at ZT12 to the Period1 promoter. Fragments with p< 0.01 are colored according to peak time in contact frequency (color-coding as in following top left circle panel D). * = local maximum in differential genomic contact. n=2. Dashed rectangle: region of rhythmic interaction. (C) 4C-seq signal over time adjacent to * (B). (D) Period1 4C-seq signal at ZT04 (purple) and ZT12 (yellow) and time-resolved ChIP-seq signals for PolII, H3K27ac and DNase1 hypersensitivity in WT mouse liver [8]. Colored tracks represent peak time in 4C-seq signal and chromatin marks following the color code as in (C) (Material and Methods). Dashed rectangle: region of rhythmic interaction. The regions located 6 kb upstream and 17 kb downstream of the bait coincided with multiple localized peaks in H3K27ac and DNase1 hypersensitivity. See S8A Fig for ChIP-seq signals of CTCF and core clock factors at the connected genomic regions. (TIFF) [file pgen.1009350.s002.tiff]

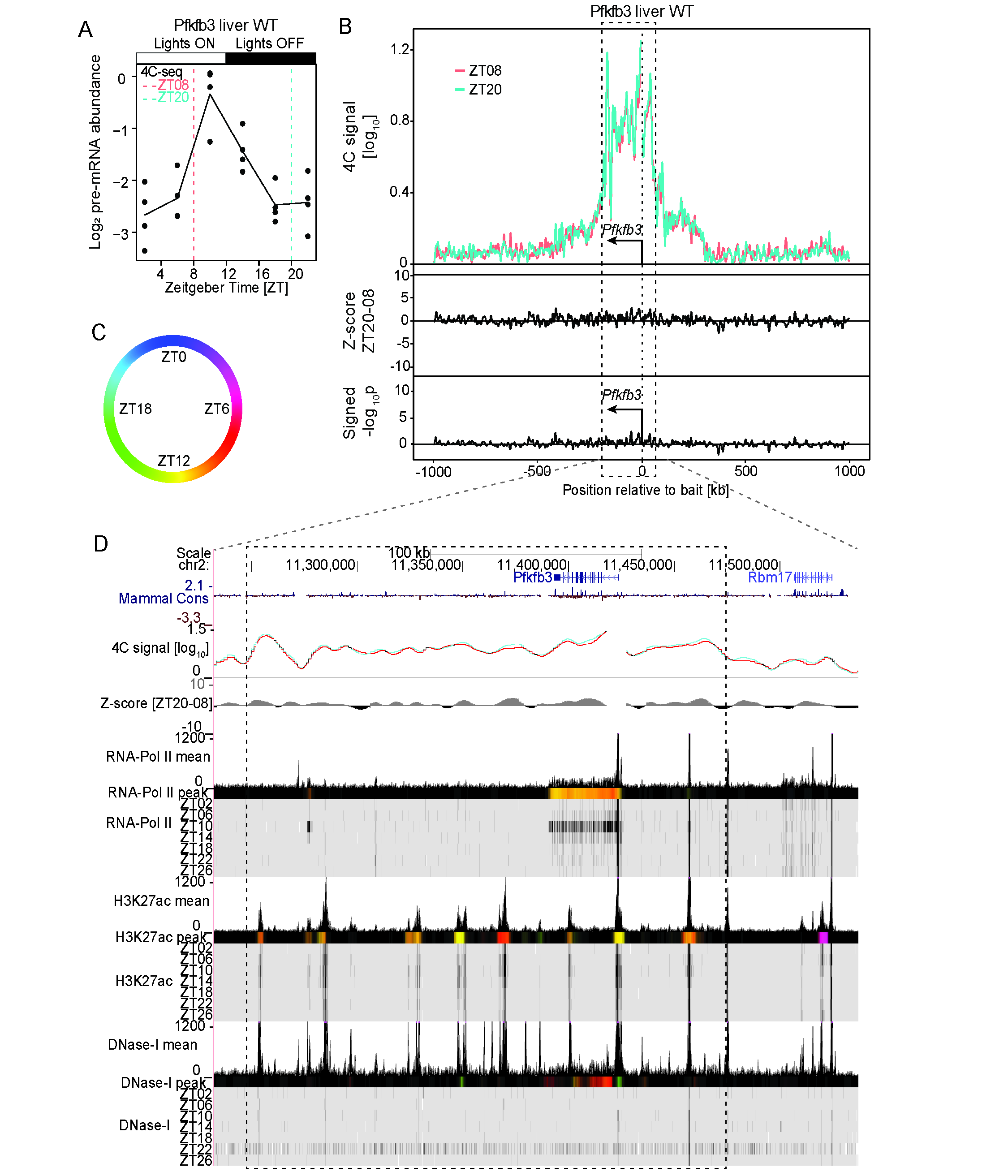

Supplement: S3 Fig — (A) Pfkfb3 pre-mRNA expression over time in WT mouse liver [47]. (B) 4C-seq signal at ZT08 (red, n=3) and ZT20 (green, n=4) in WT mouse liver in a 2Mb genomic window surrounding the Pfkfb3 bait position (upper panel) and the corresponding Z-scores (middle track) and p-values (lower track) revealing multiple prominent 4C peaks. Dashed rectangle: region of highest interaction frequency. (C and D) Genome browser viewing with Pfkfb3 4C-seq signal at ZT08 (red) and ZT20 (blue) and time-resolved ChIP-seq signal for PolII, H3K27ac and DNase1 hypersensitivity in WT mouse liver (D) [8]. Colored tracks represent peak time in chromatin marks following the color code as in (C) (Material and Methods). Dashed rectangle: region of highest interaction frequency. Multiple regions interacting with the Pfkfb3 TSS are marked by DHSs and rhythmic H3K27ac signals peaking around ZT10, in sync with Pfkfb3 transcription. See S10A Fig for ChIP-seq signals of CTCF and core clock factors at the connected genomic regions. (TIFF) [file pgen.1009350.s003.tiff]

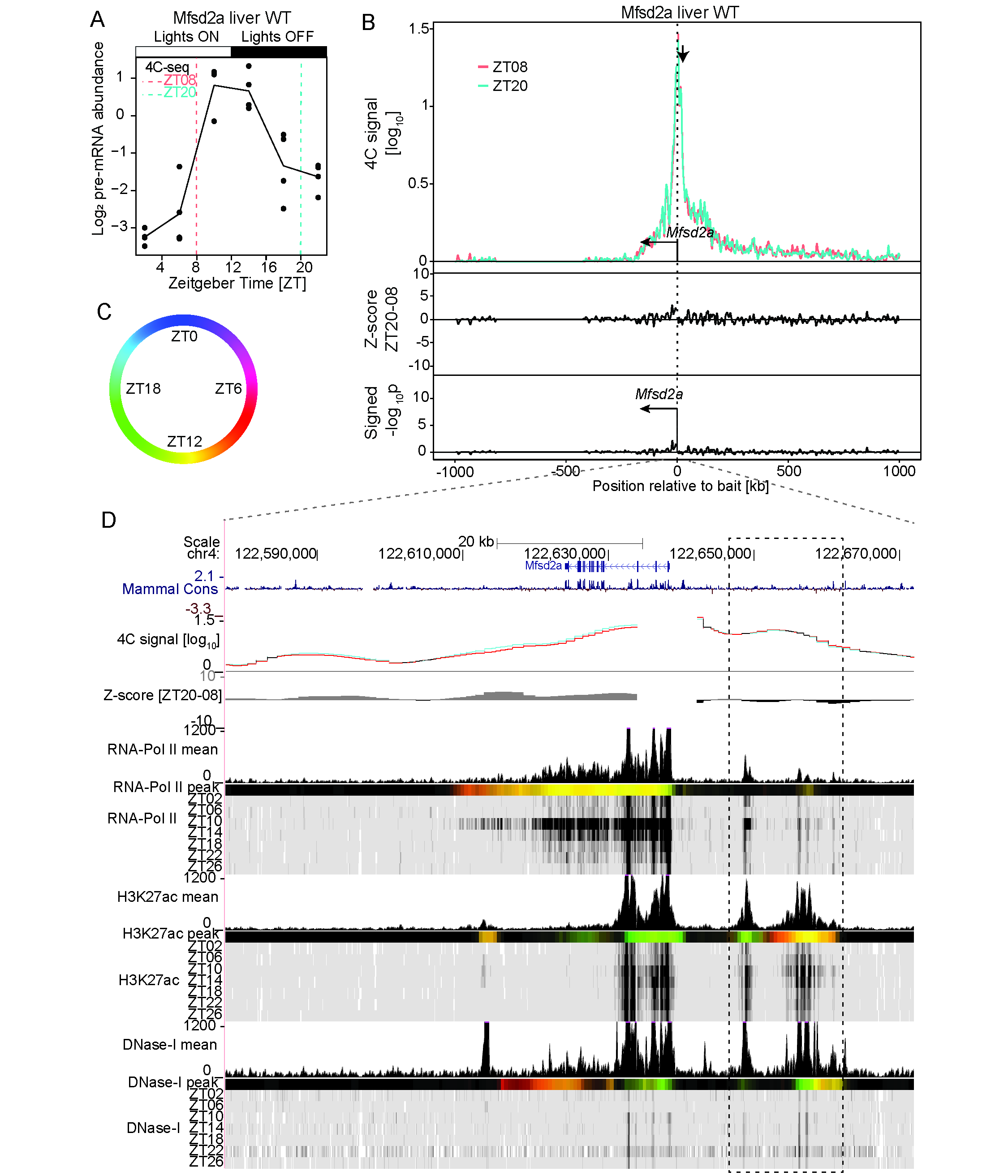

Supplement: S4 Fig — (A) Mfsd2a pre-mRNA expression over time in WT mouse liver [47]. (B) 4C-seq signal at ZT08 (red, n=3) and ZT20 (green, n=3) in WT mouse liver in a 2Mb genomic window surrounding the Mfsd2a bait position (upper panel) and the corresponding Z-scores (middle track) and p-values (lower track) revealing a localized prominent 4C-seq peak (although with low 4C-seq signals) ~15 kb upstream of the bait position (arrow). (C and D) Genome browser viewing with Mfsd2a 4C-seq signal at ZT08 (red) and ZT20 (blue) and time-resolved ChIP-seq signal for PolII, H3K27ac and DNase1 hypersensitivity in WT mouse liver (D) [8]. Dashed rectangle: region of highest interaction frequency. The genomic region located ~15 kb upstream of the bait position is marked by DHSs and rhythmic H3K27ac signals peaking around ZT12, consistently with Mfsd2a transcription. Colored tracks represent peak time in chromatin marks following the color code as in (C) (Material and Methods). See S10B Fig for ChIP-seq signals of CTCF and core clock factors at the connected genomic regions. (TIFF) [file pgen.1009350.s004.tiff]

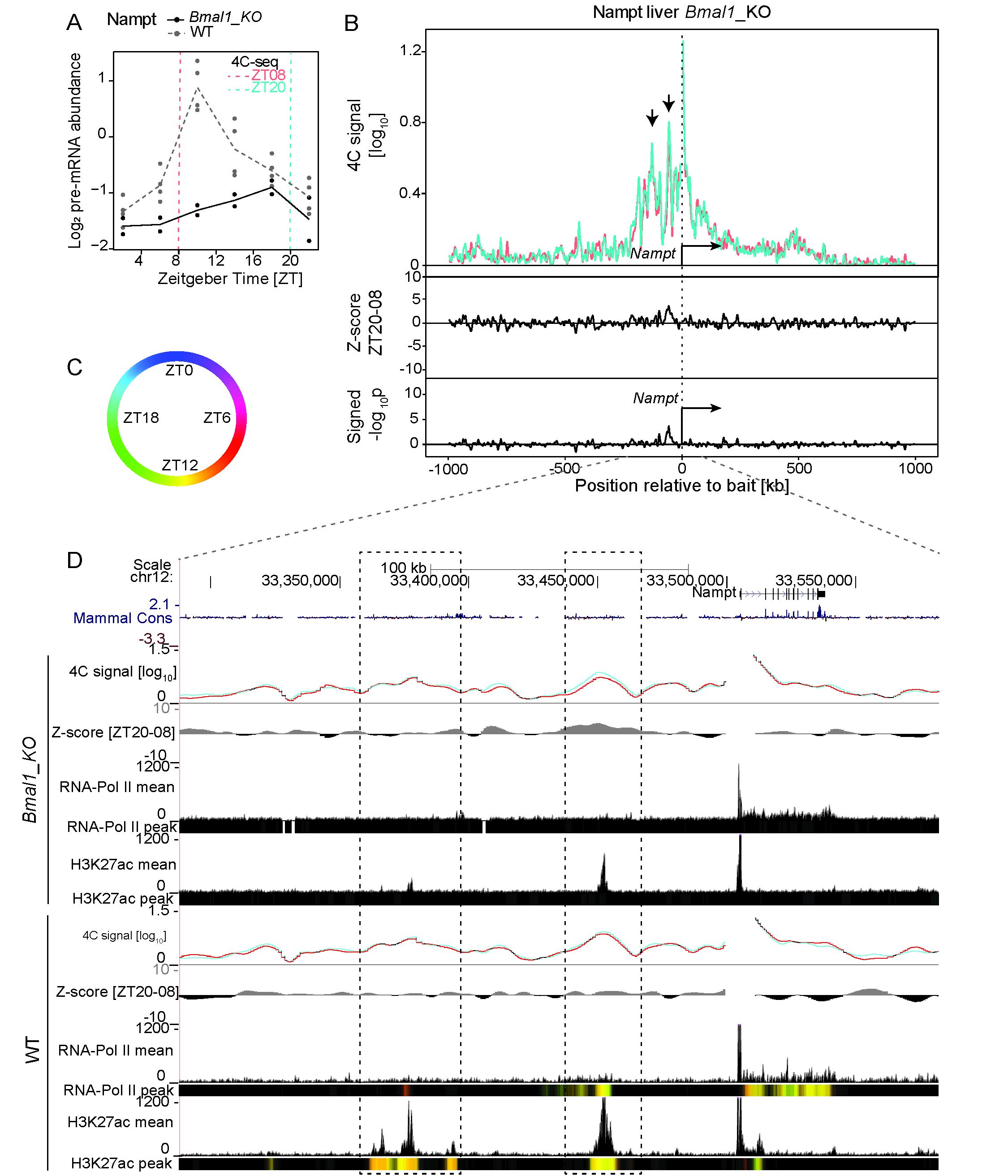

Supplement: S5 Fig — (A) Nampt pre-mRNA accumulation over time in WT (solid line) and Bmal1 KO (dashed line) livers [47]. (B) Nampt 4C-seq signal at ZT08 (red, n=3) and ZT20 (green, n=3) in a genomic window of 2Mb surrounding the bait in the liver of Bmal1 KO animals and the corresponding Z-scores and p-values. (C and D) Genome browser view of the Nampt 4C-seq signal and the corresponding Z-scores in livers of WT and Bmal1 KO animals at ZT08 (red line) and ZT20 (blue line) (D). Mean and peak time of PolII and H3K27ac ChIP-seq signals are shown for both WT and Bmal1 KO conditions (D) [8]. Colored tracks represent peak time in chromatin marks following the color code as in (C) (Material and Methods). Dashed rectangle: region of highest interaction frequency in WT and Bmal1_KO animals. H3K27ac rhythms are observed at connected regions in WT livers. In the arrhythmic Bmal1 KO livers, the chromatin marks no longer oscillate while chromatin contacts are maintained at levels comparable to WT conditions. (TIFF) [file pgen.1009350.s005.tiff]

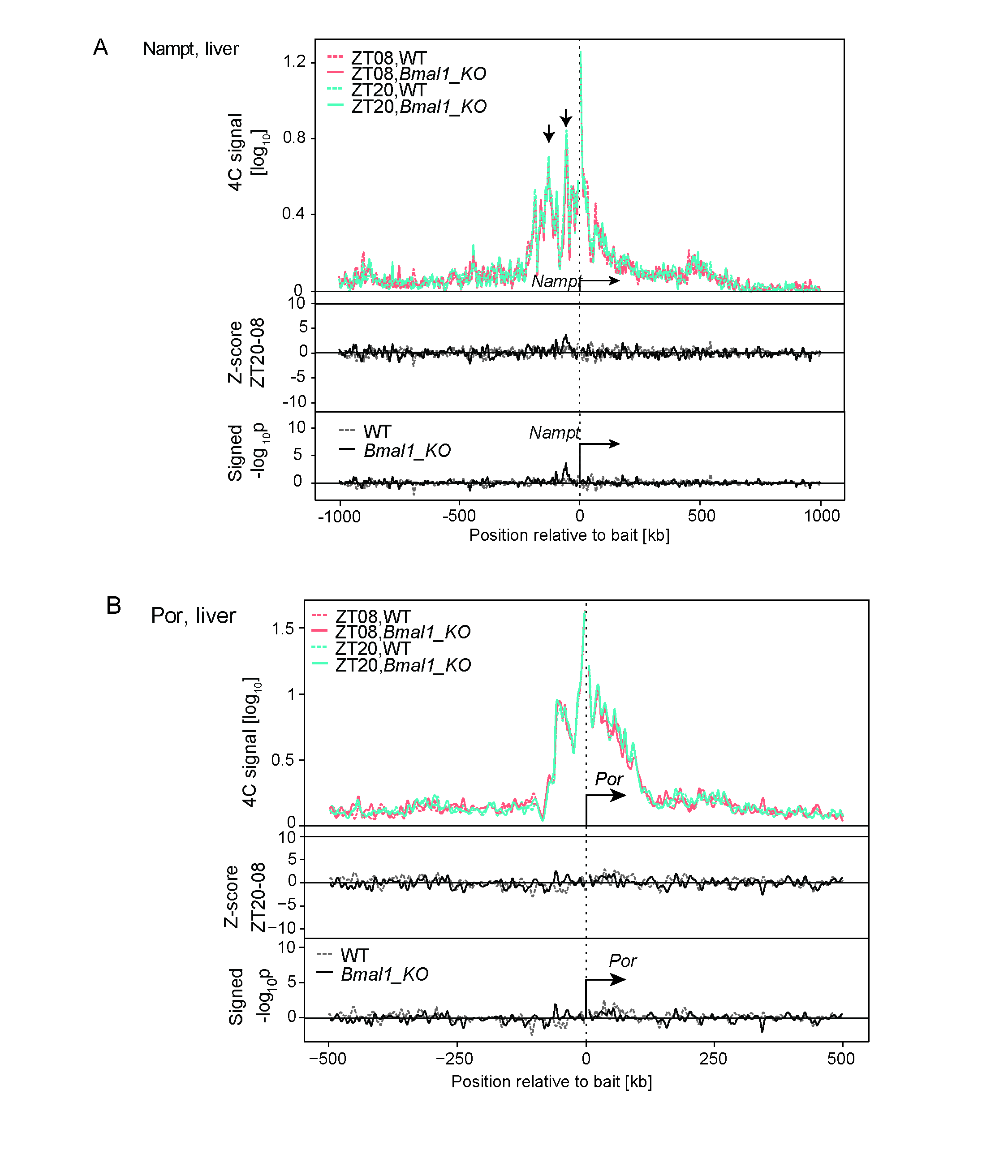

Supplement: S6 Fig — (A) Nampt 4C-seq signal at ZT08 (red) and ZT20 (green) in a genomic window of 2Mb surrounding the bait in the liver of WT (n=2 at ZT08, n=4 at ZT20, dashed lines) and Bmal1 KO animals (n=3, solid lines) and the corresponding Z-scores and p-values. Nampt 4C-seq signals are very similar across all conditions. (B) 4C-seq signal from Por TSS bait at ZT08 (red) and ZT20 (green) in a genomic window of 1Mb surrounding the bait in livers of WT (n=3 at ZT08 and n=4 at ZT20, dashed lines) and Bmal1_KO animals (n=3, solid lines) and the corresponding Z-scores and p-values. Por 4C-seq signals are very similar across all conditions. (TIFF) [file pgen.1009350.s006.tiff]

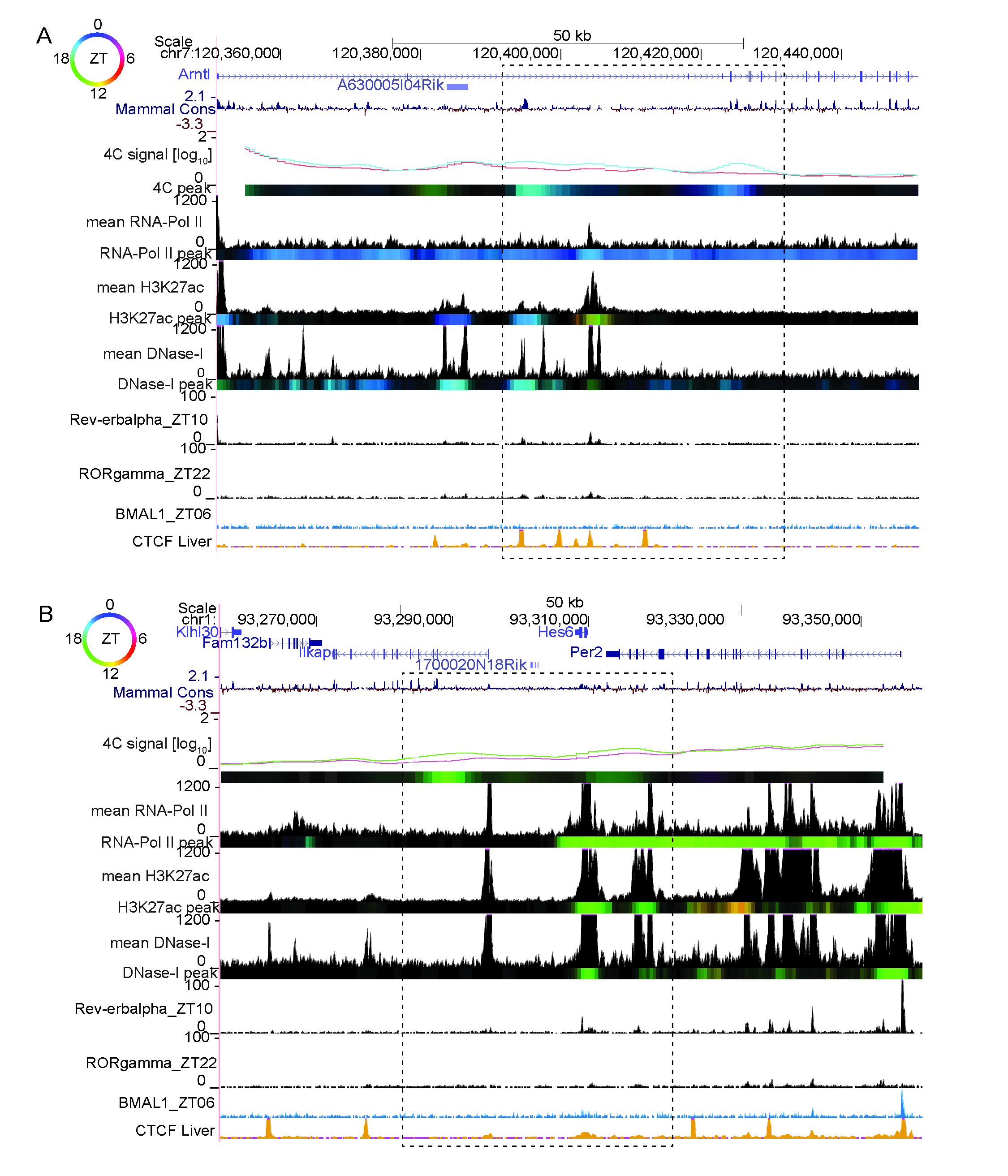

Supplement: S7 Fig — (A) 4C-seq signal and ChIP-seq signal for PolII, H3K27ac and DNase1 hypersensitivity in WT mouse liver at Bmal1 locus as in Fig 1D, as well as ChIP-seq signal against BMAL1 in mouse liver at ZT06 [6], against REVERB-alpha and ROR-gamma in mouse liver at ZT10 and ZT22 respectively [48], and CTCF in mouse liver [39]. Dashed rectangle: genomic regions contacting Bmal1 promoter preferentially between ZT18 to ZT00 are marked by synchronous rhythms in H3K27ac and DNase1 hypersensitivity as well as binding of REVERB-alpha, ROR-gamma and CTCF. (B) 4C-seq signal and ChIP-seq signal for PolII, H3K27ac and DNase1 hypersensitivity in WT mouse liver at Per2 locus as in Fig 2D, as well as ChIP-seq signal against BMAL1 in mouse liver at ZT06 [6], against REVERB-alpha and ROR-gamma in mouse liver at ZT10 and ZT22 respectively [48], and CTCF in mouse liver [39]. Dashed rectangle: genomic regions contacting Per2 promoter preferentially at ZT16 are marked by synchronous rhythms in H3K27ac and DNase1 hypersensitivity as well as binding of REVERB-alpha, ROR-gamma and CTCF, as well as low binding of BMAL1. (TIFF) [file pgen.1009350.s007.tiff]

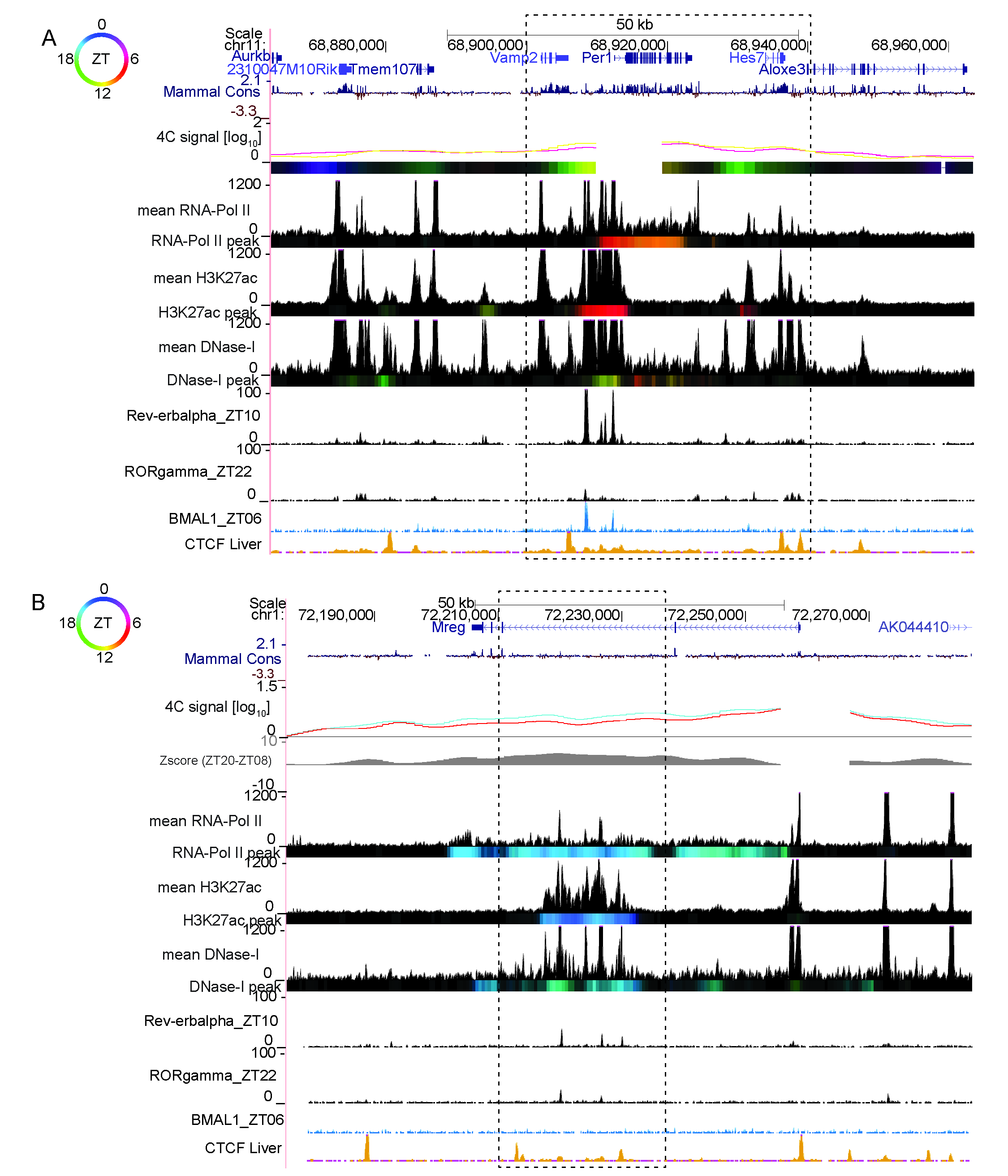

Supplement: S8 Fig — (A) 4C-seq signal and ChIP-seq signal for PolII, H3K27ac and DNase1 hypersensitivity in WT mouse liver at Per1 locus as in S2 Fig, as well as ChIP-seq signal against BMAL1 in mouse liver at ZT06 [6], against REVERB-alpha and ROR-gamma in mouse liver at ZT10 and ZT22 respectively [48], and CTCF in mouse liver [39]. Dashed rectangle: genomic regions rhythmically contacting Per1 promoter are marked by peaks of H3K27ac and DNase1 hypersensitivity as well as binding of BMAL1, REVERB-alpha, ROR-gamma and CTCF. (B) 4C-seq signal and ChIP-seq signal for PolII, H3K27ac and DNase1 hypersensitivity in WT mouse liver at Mreg locus as in Fig 3, as well as ChIP-seq signal against BMAL1 in mouse liver at ZT06 [6], against REVERB-alpha and ROR-gamma in mouse liver at ZT10 and ZT22 respectively [48], and CTCF in mouse liver [39]. Dashed rectangle: genomic regions contacting Mreg promoter preferentially at ZT20 are marked by synchronous rhythms in H3K27ac, DHS as well as binding of REVERB-alpha, ROR-gamma and CTCF. (TIFF) [file pgen.1009350.s008.tiff]

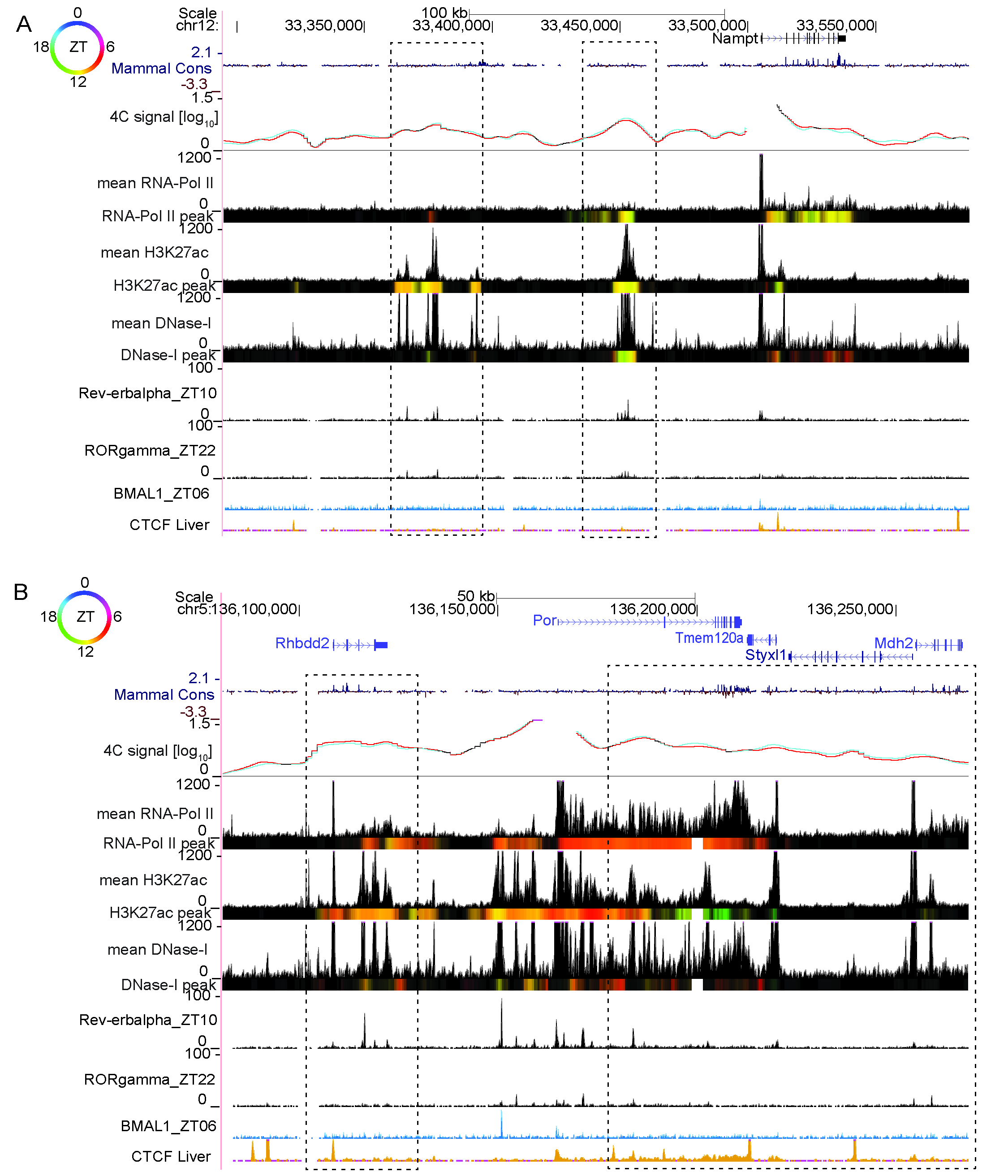

Supplement: S9 Fig — (B) 4C-seq signal and ChIP-seq signal for PolII, H3K27ac and DNase1 hypersensitivity in WT mouse liver at Nampt locus as in Fig 4, as well as ChIP-seq signal against BMAL1 in mouse liver at ZT06 [6], against REVERB-alpha and ROR-gamma in mouse liver at ZT10 and ZT22 respectively [48], and CTCF in mouse liver [39]. Dashed rectangles: genomic regions contacting Nampt promoter are marked by rhythms in PolII, H3K27ac and DNase1 hypersensitivity as well as binding of REVERB-alpha and ROR-gamma. (B) 4C-seq signal and ChIP-seq signal for PolII, H3K27ac and DNase1 hypersensitivity in WT mouse liver at Por locus as in Fig 5, as well as ChIP-seq signal against BMAL1 in mouse liver at ZT06 [6], against REVERB-alpha and ROR-gamma in mouse liver at ZT10 and ZT22 respectively [48], and CTCF in mouse liver [39]. Dashed rectangles: genomic regions contacting Por promoter are marked rhythms in PolII, H3K27ac and DNase1 hypersensitivity as well as binding of REVERB-alpha, ROR-gamma and CTCF. (TIFF) [file pgen.1009350.s009.tiff]

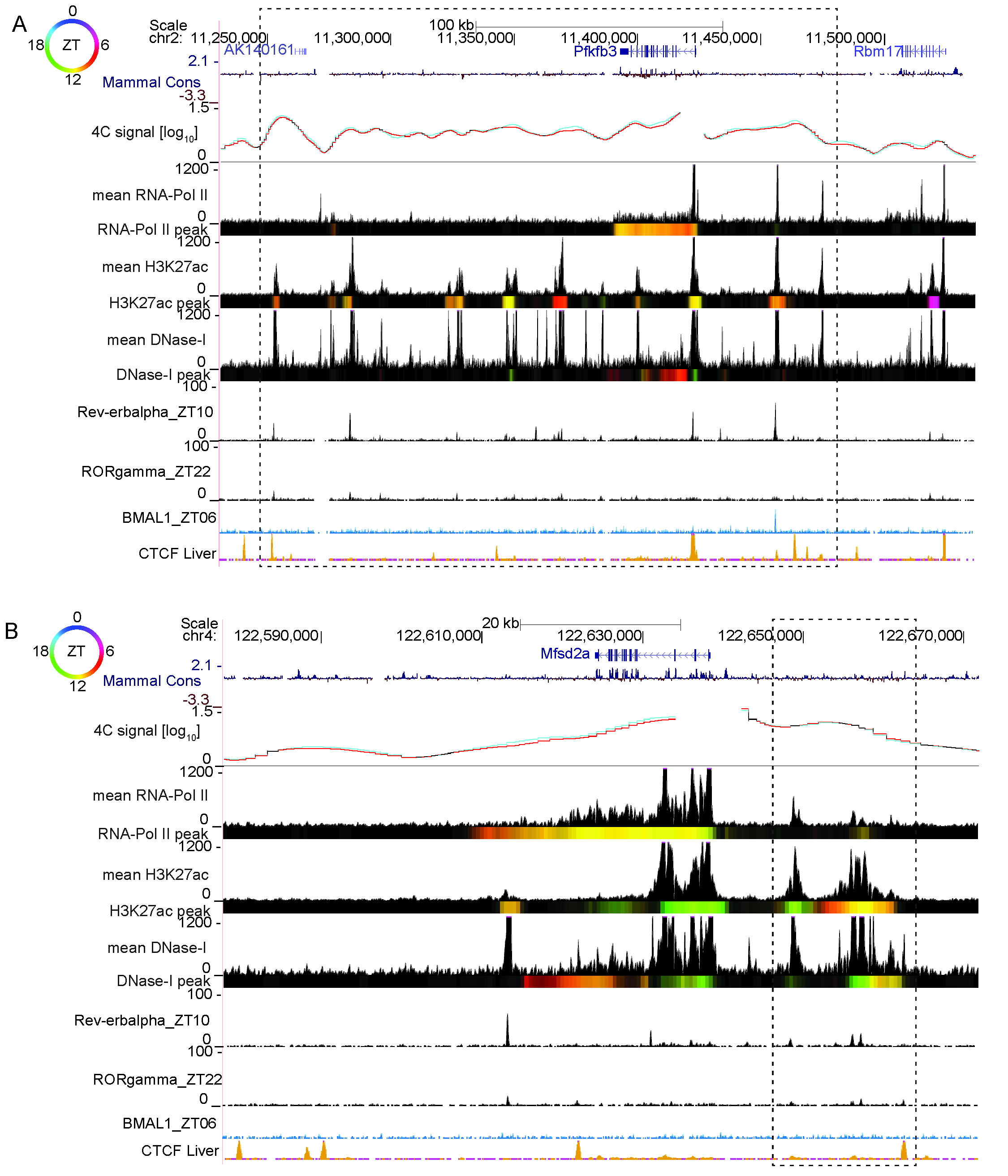

Supplement: S10 Fig — (A) 4C-seq signal and ChIP-seq signal for PolII, H3K27ac and DNase1 hypersensitivity in WT mouse liver at Pfkfb3 locus as in S3D Fig, as well as ChIP-seq signal against BMAL1 in mouse liver at ZT06 [6], against REVERB-alpha and ROR-gamma in mouse liver at ZT10 and ZT22 respectively [48], and CTCF in mouse liver [39]. Dashed rectangle: genomic regions contacting Pfkfb3 promoter are marked by rhythms in H3K27ac and DNase1 hypersensitivity as well as binding of BMAL1, REVERB-alpha, ROR-gamma and CTCF. (B) 4C-seq signal and ChIP-seq signal for PolII, H3K27ac and DNase1 hypersensitivity in WT mouse liver at Mfsd2a locus as in S4D Fig, as well as ChIP-seq signal against BMAL1 in mouse liver at ZT06 [6], against REVERB-alpha and ROR-gamma in mouse liver at ZT10 and ZT22 respectively [48], and CTCF in mouse liver [39]. Dashed rectangle: genomic regions contacting Mfsd2a promoter are marked rhythms in PolII, H3K27ac and DNase1 hypersensitivity as well as binding of REVERB-alpha and ROR-gamma, and low binding of CTCF. (TIFF) [file pgen.1009350.s010.tiff]

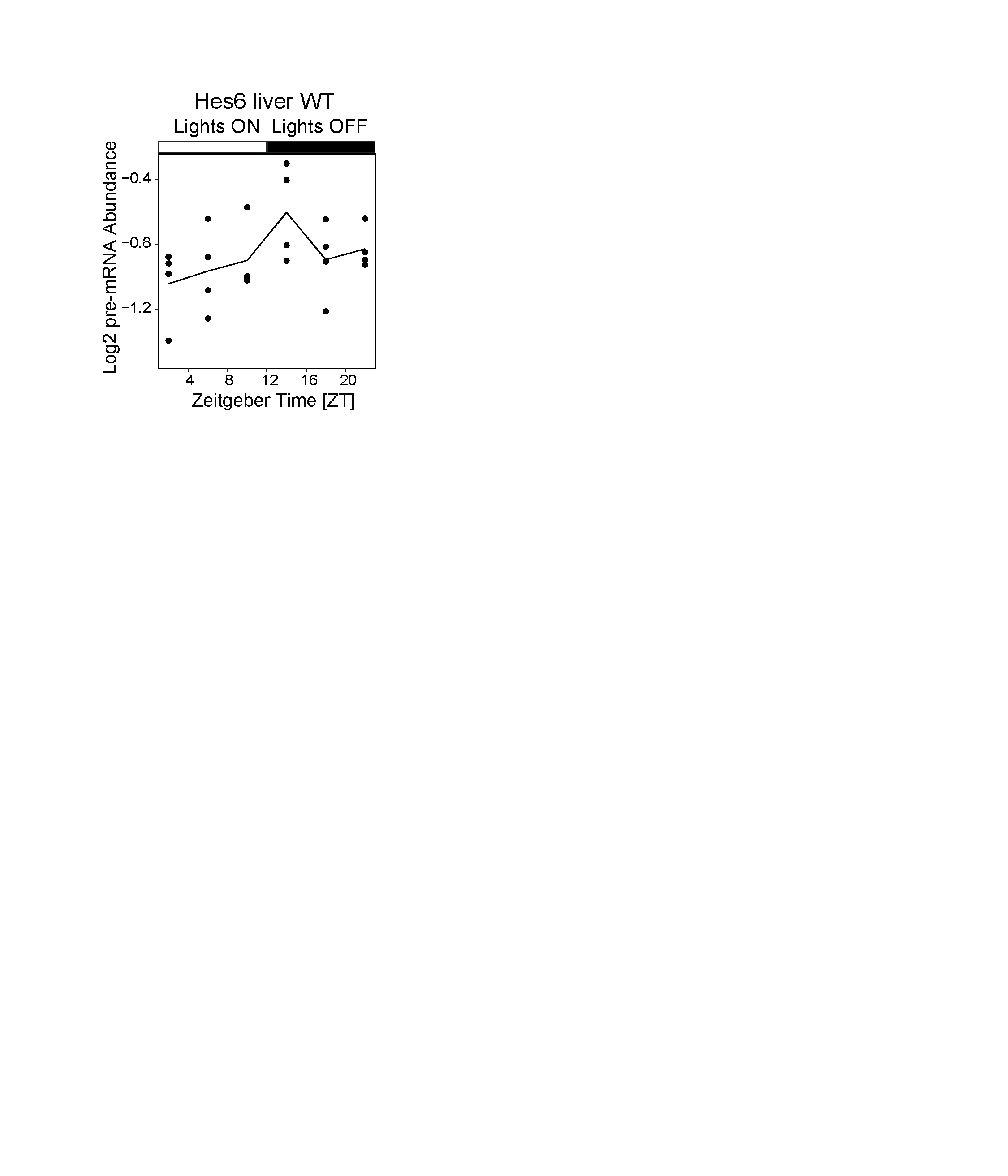

Supplement: S11 Fig — Data from [47]. (TIFF) [file pgen.1009350.s011.tiff]
